# RNY-derived small RNAs as a signature of coronary artery disease

**DOI:** 10.1186/s12916-015-0489-y

**Published:** 2015-10-08

**Authors:** Emanuela Repetto, Laeticia Lichtenstein, Zoheir Hizir, Nedra Tekaya, Mohamed Benahmed, Jean-Bernard Ruidavets, Laure-Emmanuelle Zaragosi, Bertrand Perret, Laura Bouchareychas, Annelise Genoux, Romain Lotte, Raymond Ruimy, Jean Ferrières, Pascal Barbry, Laurent O. Martinez, Michele Trabucchi

**Affiliations:** INSERM U1065 and University of Nice Sophia Antipolis, Centre Méditerranéen de Médecine Moléculaire (C3M), Team 10 “Control of Gene Expression”, F-06204 Nice, France; INSERM UMR 1048, Toulouse, 31000 France; INSERM U1065, Team 5, F-06204 Nice, France; INSERM U1027, Faculté de Médecine, Toulouse, 31073 France; CNRS and University of Nice Sophia Antipolis, IPMC, Sophia Antipolis, Nice, France; Université de Toulouse III, UMR 1048, Toulouse, 31300 France; CHU de Toulouse, Hôpital Purpan, Toulouse, France; Sorbonne Universités, UPMC Université Paris 06, UMR_S 1166, ICAN, Integrative Biology of Atherosclerosis Team, Paris, F-75005 France; Hôpital Archet, Nice, France

**Keywords:** Atherosclerosis, Biomarker, Coronary artery disease, Macrophage, Small RNA

## Abstract

**Background:**

Data from next generation sequencing technologies uncovered the existence of many classes of small RNAs. Recent studies reported that small RNAs are released by cells and can be detected in the blood. In this report, we aimed to discover the occurrence of novel circulating small RNAs in coronary artery disease (CAD).

**Methods:**

We used high-throughput sequencing of small RNAs from human and mouse apoptotic primary macrophages, and analyzed the data by empirical Bayes moderated t-statistics to assess differential expression and the Benjamini and Hochberg method to control the false discovery rate. Results were then confirmed by Northern blot and RT-qPCR in foam cells and in two animal models for atherosclerosis, namely *ApoE*^*−/−*^ and *Ldlr*^*−/−*^ mouse lines. Quantitative RT-PCR to detect identified small RNAs, the RNY-derived small RNAs, was performed using sera of 263 patients with CAD compared to 514 matched healthy controls; the Student *t*-test was applied to statistically assess differences. Associations of small RNAs with clinical characteristics and biological markers were tested using Spearman’s rank correlations, while multivariate logistic regressions were performed to test the statistical association of small RNA levels with CAD.

**Results:**

Here, we report that, in macrophages stimulated with pro-apoptotic or pro-atherogenic stimuli, the Ro-associated non-coding RNAs, called RNYs or Y-RNAs, are processed into small RNAs (~24–34 nt) referred to as small-RNYs (s-RNYs), including s-RNY1-5p processed from RNY1. A significant upregulation of s-RNY expression was found in aortic arches and blood plasma from *ApoE*^*−/−*^ and *Ldlr*^*−/−*^ mice and in serum from CAD patients (*P* <0.001). Biostatistical analysis revealed a positive association of s-RNY1-5p with hs-CRP and ApoB levels; however, no statistical interaction was found between either of these two markers and s-RNY1-5p in relation to the CAD status. Levels of s-RNY1-5p were also independent from statin and fibrate therapies.

**Conclusion:**

Our results position the s-RNY1-5p as a relevant novel independent diagnostic biomarker for atherosclerosis-related diseases. Measurement of circulating s-RNY expression would be a valuable companion diagnostic to monitor foam cell apoptosis during atherosclerosis pathogenesis and to evaluate patient’s responsiveness to future therapeutic strategies aiming to attenuate apoptosis in foam cells in advanced atherosclerotic lesions.

**Electronic supplementary material:**

The online version of this article (doi:10.1186/s12916-015-0489-y) contains supplementary material, which is available to authorized users.

## Background

There are several classes of small RNAs, including microRNAs, siRNAs, piRNAs, and those derived from sno-RNAs, tRNAs, or Alu repeats. Significant amounts of microRNAs have been recently found in extracellular body fluids such as blood, urine, saliva, and semen [1]. Some circulating microRNAs in the blood have been proposed as biomarkers for several human disorders, including cardiovascular diseases [1, 2].

In this report, we have explored whether pro-apoptotic stimuli regulate the expression of novel small RNAs in macrophages using a high-throughput sequencing approach. Macrophage apoptosis is a tightly-regulated mechanism that controls tissue homeostasis and can be induced by a variety of signals and stress factors, including bacterial toxins, DNA damage, oxidant stress, endoplasmic reticulum (ER) stressors, cytokines, activation of Fas death pathway, modified low-density lipoprotein (LDL) such as oxidized LDL (oxLDL) or acetylated LDL (acLDL), and saturated fatty acids [3, 4]. Its physiopathological importance is highlighted by the fact that apoptosis in macrophages is involved in many inflammatory diseases, including atherosclerosis [4]. In particular, induction of macrophage apoptosis accelerates the development of atherosclerosis in advanced atherosclerotic lesions [5, 6].

A key initiating step of atherogenesis is the subendothelial accumulation of apolipoprotein B (apoB)-containing lipoproteins (apoB-LPs), mainly composed by LDL [7]. The modification of LDL lipid content triggers a low-grade inflammatory response leading to activation of endothelial and vascular smooth muscle cells (SMCs) and recruitment of monocytes and monocyte-derived macrophages, which play a major and dual role in atherosclerosis pathogenesis [4]. In fact, in early phases, macrophages engulf LDL and modified LDL, becoming lipid-laden macrophages – so-called foam cells, which secrete inflammatory cytokines and undergo apoptosis. Rapid efferocytic clearance of apoptotic foam cells leads to a decrease of the inflammatory response and retards atherosclerosis progression [8]. However, in advanced atherosclerotic lesions, macrophage apoptosis is not properly coupled with phagocytic clearance, leading to an upregulation of inflammation and to necrotic plaque formation [9]. This may ultimately lead to clinical events such as heart attack or stroke [10]. Atherosclerosis is responsible for cardiovascular diseases, including coronary artery disease (CAD), peripheral vascular diseases, and strokes, which are now considered to be the single largest scourge of modern society [10]. CAD occurs when atherosclerosis affects the heart arteries. According to the World Health Organization, in 2008, nearly 30 % of deaths worldwide were attributed to cardiovascular diseases, which are predicted to become the greatest single-disease cause of death at a global scale by 2030 [11]. This will ultimately lead to an increase in direct and indirect costs in the treatment of CAD patients, which was estimated to be about $ 165 billion in the United States alone in 2009 [11]. Although assessment of classic cardiovascular risk factors or markers, including high blood pressure, diabetes, smoking, and high LDL-cholesterol, has had a central role in disease prevention and therapeutic choice, the need of novel diagnostic biomarkers for atherosclerosis is underlined by the fact that even the reduction of classical risk factor and/or marker levels, such as LDL-cholesterol, does not fully prevent coronary events, which still occur at high rates [12]. In addition, a great clinical need exists for biomarkers for risk stratification to restrict current therapies to patients at greatest risk of clinical events, who most likely will benefit the most. On the other hand, it would reduce side effects and unnecessary costs among patients at low risk, for whom the benefit-risk balance of therapies is less favorable [7].

Herein, we demonstrate that the ~110 nucleotide (nt) long Ro-associated non-coding RNAs, termed RNYs, are processed into small 24–34 nt sequences in macrophages upon pro-apoptotic or pro-atherogenic treatments. The expression of RNY-derived small RNAs is significantly upregulated in two mouse models for atherosclerosis and in the serum of CAD patients. Our data indicate that RNY-derived small RNAs can be used as novel independent biomarkers, related to apoptosis of lesional macrophages, well suited for the assessment of CAD risk or in the setting of future therapeutic strategies aiming to attenuate macrophage apoptosis in advanced atherosclerotic lesions.

## Methods

### Small RNA sequencing: experimental design

Human primary macrophages were treated with 1 μM staurosporine (STS) for 4 or 6 h, whereas mouse bone marrow-derived macrophages (BMDMs) were incubated in a Macrophage-Colony Stimulating Factor (M-CSF) withdrawal conditioned medium for 36 h. We then extracted total RNA from treated and untreated cells using RNA columns (miRNeasy, Qiagen). The RNA quality was checked on a bioanalyzer with the RNA 6000 Nano kit (Agilent Technologies). Two small RNA sequencing runs were performed. In the first, 1.5 μg of total RNA from one biological replicate for each condition were subjected to small RNA library preparations using the NEBNext Small RNA Library Prep Set for SOLID™ kit (New England Biolabs), following the manufacturer’s instructions. Libraries were then sequenced on a SOLiD 5500 platform (Life Technologies) with a 35-bp fragment length. For the second run, 50 ng of total RNA from two biological replicates for each condition were subjected to small RNA library preparation with the CleanTag library kit (TriLink Biotechnologies) following the manufacturer’s instructions. These libraries were sequenced on an Ion Proton PI Chip (Life Technologies).

Two NEBNext Small RNA libraries were also made from 25–40 nt size selected RNA purified by polyacrylamide gel electrophoresis from M-CSF withdrawal-treated mouse macrophages and control, and sequenced with SOLiD 5500 platform to control the presence of cleaved forms of RNYs.

### Small RNA sequencing: data analysis

Fragments that were sequenced with SOLiD run were mapped on hg19 for human samples and mm10 for mice samples within LifeScope 2.5.1 small RNA pipeline (Life Technologies) with default parameters; .bam files were then merged with MergeSamFiles.jar (picard-tools-1.38) for each sample, keeping a read group per lane. Fragments that were sequenced with Ion Proton P1 Chip were first trimmed with cutadapt v1.2.1 for 5p adapter TTCTACAGTCCGACGATC and 3p adapter TGGAATTCTCGGGTGCCAAGG. Reads with size <15 bases were discarded. Remaining reads were mapped to hg19 build for human samples and mm10 build for mouse samples with bowtie2 (v2.2.4) with “--local --very-sensitive-local -k 10”. All .bam files were processed with a java homemade class, based on Picard java classes to identify and quantify small RNAs. Annotation files used for quantification correspond to miRbase release 21, tRNAs from UCSC web server (UCSC tables), RNYs and ncRNAs from ensembl ncRNAs release 75, and piRNAs from fRNAdb.

All sequencing results were submitted to the GEO database under the superseries accession number GSE63802.

For quantitative analysis of small RNAs, each count data table generated from SOLiD and Ion Proton experiments was merged based on feature IDs. When the sum of reads mapped on each gene was less than five reads in a specific experiment, they were discarded (low level of expression). The final datasets comprised 1,338 genes for mouse data and 1,435 genes for human data. Functions from the Bioconductor package limma [13] were used to normalize data and assess differential expression.

For statistical analysis, count data were first normalized for sequencing depth differences and transformed into log2 count per million using the voom function [14] and differential expression was assessed using linear modeling. For each gene, a linear model was fit with two parameters, one for the comparison of interest (treatment vs. control) and one to account for potential technology bias between the two runs. Empirical Bayes moderated t-statistics were used to assess differential expression. The Benjamini and Hochberg method was used to control the false discovery rate [15], for which a value <0.05 was considered significant.

### Animals and diets

The following mice were purchased from Charles River Laboratories (L’Arbresle, France): C57BL/6 J, *ApoE*^*−/−*^ (B6.129P2-APOE/J), and *Ldlr*^*−/−*^ (B6.129S7-Ldlr^tm1Her^/J). High cholesterol diet (HCD) formula # TD02028 (1.3 % of cholesterol) and TD96335 (1.25 % of cholesterol) for *ApoE*^*−/−*^ and *Ldlr*^*−/−*^, respectively, were purchased from ssniff Spezialdiaten GmbH (Soest, Germany). *ApoE*^*−/−*^ and *Ldlr*^*−/−*^ male mice at 8 weeks of age were fed with either HCD or regular diet (chow diet) for 12 and 20 weeks, respectively. Aortic arches, femoral arteries, and blood plasma were dissected. *CD68-hBcl-2 ApoE*^*−/−*^ was generated as previously described [5]. Blood plasma of mice fed with chow diet was collected at the age of 27–30 weeks.

### Reagents

Lipoteichoic acid from the Gram-positive bacteria *Staphylococcus aureus*, thapsigargin (Tg), STS, bovine serum albumin, palmitic acid, stearic acid, oleic acid, linoleic acid, fluvastatin, simvastatin, lovastatin, and pravastatin were purchased from Sigma (Saint-Quentin Fallavier, France). oxLDL was purchased from Clinisciences (Nanterre, France). LDL was isolated from the plasma of normolipidemic healthy human donors by standard sequential ultracentrifugation of discontinuous KBr gradients. LDL were acetylated (acLDL) by addition of 1.5 μL acetic anhydride per milligram of LDL in 50 % ice-cold saturated sodium acetate over a 1-hour period at 4 °C. The acLDL was dialyzed against 150 mM NaCl, 5 mM Tris–HCl, and 0.3 mM EDTA, pH 7.4 at 4 °C and gave a single band on agarose electrophoresis. Anti-Ro60 (TROVE2) and anti-Argonaute 2 antibodies were purchased from GmbH and Wako, respectively.

### Primary cells and cell lines

Bone marrow cells were collected from femurs and tibias of 10-week-old male C57BL/6 J mice by flushing with sterile medium as previously described [16]. To differentiate into macrophages, bone marrow cells (×10^6^) were plated in 10-cm plates in 7 mL of BMDM medium (Dulbecco’s Modified Eagle Medium (DMEM) supplemented with 20 % low-endotoxin fetal bovine serum, 30 % L929-cell conditioned medium, 1 % l-glutamine, 1 % Pen/Strep, 0.5 % Na pyruvate, and 0.1 % β-mercaptoethanol), and fed with 2.5 mL of fresh medium every two days for 7 days. Human primary macrophages were prepared from peripheral blood monocytes obtained from healthy donors [17] in agreement with the French legislation on human biomedical research (all participants provided written informed consent attesting they had received all the information required about the study and they agreed to its publication in accordance with appropriate national regulations).

Mouse aortic vascular smooth muscle cells (ATCC® CRL-2797 cell line) and murine endothelial cells (ATCC® CRL 2181 cell line) were seeded in six-well plates (2 × 10^6^ and 2 × 10^5^ cells/mL, respectively) and grown in DMEM, high glucose, GlutaMAX™ (Life Technologies) with 10 % heat-inactivated fetal bovine serum, 100 IU/mL penicillin, and 100 μg/mL streptomycin (Life Technologies). Adult ventricular myocytes were obtained from the heart of C57/BL6 male mice at 8 weeks of age with retrograde perfusion as previously described [18]. The cell pellet was resuspended in DMEM containing 2 mg/mL bovine serum albumin, 2 mmol/L l-carnitine, 5 mmol/L creatine, 5 mmol/L taurine, 100 IU/mL penicillin, and 100 μg/mL streptomycin. Isolated myocytes were then seeded onto gelatin-coated six-well plates (1 × 10^6^ cells/mL) in Ham-F12 medium supplemented with 3 % fetal bovine serum.

### Cell transfection and RNA immunoprecipitation

BMDMs were transiently transfected for 48 h with Lipofectamine 2000 (Invitrogen) according to the manufacturer’s instructions. 2′-O-Me-RNA antisense oligonucleotides to s-RNYs (Eurogentec) were transfected at the final concentration of 100 nM. A total of 300 μg of proteins was immunoprecipitated with Protein A-Dynabeads-coupled (Invitrogen) antibodies for 16 h at 4 °C with rotation. Immunoprecipitated pellets were washed four times with lysis buffer and resuspended in Trizol (Invitrogen), then analyzed by RT-qPCR. The primer sequences are detailed in Additional file 1: Table S6.

### Northern blot

Total RNA was isolated from cells using Trizol (Invitrogen), resolved on 10 % polyacrylamide-urea gels, and electroblotted onto HyBond N+ membranes. Membranes were hybridized overnight with radiolabeled DNA antisense oligonucleotides to s-RNYs in ExpressHyb solution (Clontech). After hybridization, membranes were washed three times with 2× SSC and 0.05 % SDS, twice with 0.1× SSC and 0.1 % SDS, and exposed overnight onto a film. The same blot was hybridized (upon stripping in boiling 0.1 % SDS) up to three distinct probes, including control U6 snRNA (Additional file 1: Table S6).

### s-RNY expression analysis and cloning

For s-RNYs detection by quantitative RT-PCR we used the stem-loop quantitative RT-PCR method previously described by Chen et al. [19]. This method allows the detection of only s-RNYs derived from the 5′ end of the precursor by quantitative RT-PCR analysis, namely the s-RNY1-5p and s-RNY3-5p from mouse and s-RNY1-5p, s-RNY3-5p, and s-RNY4-5p from human. Briefly, total RNA was isolated from cells, tissue, or immunoprecipitation using Trizol (Invitrogen) according to the manufacturer’s instructions.

RNA extraction from extracellular medium, blood plasma, and serum was performed as previously described [1]. Briefly, before the extraction of RNA, we checked hemolysis by spectrophotometrically measuring oxyhemoglobin at 414 nm and referring to a standard curve made by introducing hemolysis by serially diluting lysed red blood cells in non-hemolysed plasma from a healthy donor [20]. The serum samples showing an optical density corresponding to more than 0.002 % of red blood cells (v/v) were discarded. Then, 0.6 mL of Trizol LS were added to 0.2 mL of blood plasma, serum, or media. Following this, (1) 5 pg of synthetic miRNA-39 from *Caenorhabditis elegans* (cel-miR-39) were added as a spike-in control for purification efficiency and (2) 1.2 μL of glycogen (10 mg/mL) were added to enhance the efficiency of RNA column binding. Purification of extracted total RNA was performed with miRNeasy columns (Qiagen) according to the manufacturer’s instructions. RNA was eluted in 30 μL of RNase-free water and subjected to on-column DNase I treatment with RNase-free DNase (Qiagen). The quality of the extracted RNA was checked by ratio between the absorbance values at 260 and 280 nm and between 260 and 230 nm using a NanoDrop Technologies ND-1000 spectrophotometer. As cell-free RNA, such as that from serum or the extracellular milieu, does not contain any ribosomal RNAs, we therefore checked the presence of ribosomal RNAs in some samples by pico total RNA bioanalyzer (Agilent) as a sign of cellular contamination.

Reverse transcriptase reaction was performed according to Chen et al. [19] for the detection of s-RNYs, n-code (Life Technologies) for RNU48, and TaqMan (Life Technologies) for the miRNAs. Quantitative RT-PCRs using Sybr Green or TaqMan (Invitrogen) for s-RNYs, cel-miR-39, miR-24, miR-17, miR-92a, miR-126, miR-133, miR-145, miR-155, RNU48, and miR-208 were performed on a StepONE system (Applied Biosystem). Expression was considered undetectable with Ct value ≥40. The target small RNA expression value was normalized with a reference gene: the exogenous spike-in cel-miR-39, or the endogenous hsa-miR-24 [21] or RNU48 [22], as indicated in the respective legends. The relative expression level of s-RNYs was then further normalized by the 2^-ΔΔCt^ method. The Student *t*-test was performed to assess statistical significance. The primer sequences are detailed in Additional file 1: Table S6.

To assure that the small-RNAs detected by RT-qPCR were indeed derived from RNYs, we immunoprecipitated Ro60 with an anti-Ro60 antibody followed by RT-qPCR (Additional file 2: Figure S3D), showed that there was a lack of signal in palmitic acid-treated macrophages transfected with 2′-O-Me-RNA antisense oligonucleotides to s-RNYs (Additional file 3: Figure S5A), and cloned the RT-PCR products from CAD patients in pCR4-TOPO vector according to the manufacturer’s instructions (Additional file 3: Figure S5B; other data not shown). Altogether, these data indicate that the s-RNYs detected were indeed derived from RNYs.

### Study sample

The Génétique et Environnement en Europe du Sud (GENES) study is a case–control study designed to investigate the role of genetic, biological, and environmental determinants in the occurrence of CAD. As previously described [23, 24], cases were stable CAD patients, aged 45–74, living in the Toulouse area (southwest France) and prospectively recruited from 2001 to 2004 after admission to the Cardiology Department, Toulouse University Hospital, for cardiovascular examination and referred for evaluation and management of their CAD. Stable CAD was defined by a previous history of acute coronary syndrome, a previous history of coronary artery revascularization, a documented myocardial ischemia, a stable angina, or the presence at coronary angiography of a coronary stenosis of 50 % or more. Patients who had presented an acute coronary episode during the past 8 days were not included in the study, because they were considered unstable. During the same period, male controls, aged 45–74, were selected from the general population using electoral rolls. Stratification into decadal age groups was used to approximately match the age distribution of the people with and without CAD.

In the present study, s-RNY1-5p expression was analyzed in 263 randomly selected CAD patients from the initial sample; 514 control subjects were frequency-matched to the cases with respect to age in a 2:1 ratio. This cohort was subsequently randomly divided into one derivation sub-cohort of 43 CAD patients and 106 controls, and one validation sub-cohort of 220 CAD patients and 408 controls. Male controls underwent medical examination in the same health center administered by the national health insurance system, located approximately in the center of the study area. Similarly, male cases were examined at the Department of Cardiology, Toulouse University Hospital, over the same period. Inclusion commenced after the provision of oral and written information about the aim of the study, followed by the signing of informed consent. The medical examination took approximately 120 minutes. Medical examination included clinical and anthropometric measurements and was followed by the completion of a questionnaire (lasting for approximately 40 minutes). Blood samples were taken and a blood sample collection was constituted.

Serum was collected from 17 male individuals infected from different portal of entries (lung, bone, cutaneous, digestive, urinary tract) by various bacteria (Additional file 4: Table S5). No clinical evidence of atherosclerotic lesions was reported on this cohort. s-RNY1-5p and high sensitive C-reactive protein (hs-CRP) expression was measured and compared to 35 age-matched controls from the GENES study.

### Data collection

Male participants with and without CAD were examined in the morning after an overnight fast. Patients with stable CAD (cases) were examined in the clinics of the Cardiology Department, Toulouse University Hospital, and underwent a coronary angiography. Control subjects were examined in the same health center. The medical examination started with standardized face-to-face interviews performed by a trained physician using standardized methods. During this interview, participants received oral and written information on the aim of the study and signed a consent form. Then, a blood sample was taken from each participant. Each participant then completed standardized questionnaires, covering age, socioeconomic variables, educational level (number of years spent at school), smoking status, alcohol consumption, physical activity, information on cardiovascular risk factors, and past medical (personal and parental) history. The medical examination was continued with anthropometric and clinical measurements. Tobacco consumption was expressed in pack-year by multiplying the number of packs of cigarettes smoked per day by the number of years the person had smoked. Alcohol consumption was assessed using typical weekly patterns. Physical activity was investigated through a standardized questionnaire [25] and categorized into three levels: no physical activity; moderate physical activity, over 20 minutes no more than once a week; and high physical activity, over 20 minutes, at least twice a week. Presence of dyslipidemia, diabetes, or hypertension was assessed from the subjects’ current treatments. In those with CAD, their medication at discharge was also considered. Anthropometrical measurements included waist circumference and body mass index (BMI). Blood pressure and heart rate were measured with an automatic sphygmomanometer. Right arm blood pressure was measured twice, and the average value was recorded. Measurements were performed after a 5 min rest. The ankle-arm index was determined as previously described, and a value ≤0.9 was considered abnormal [26]. Blood samples were analyzed for serum total cholesterol, HDL-cholesterol, triglycerides, glucose, γ-glutamyltransferase, and hs-CRP with enzymatic reagents on an automated analyzer (Hitachi 912, Roche Diagnostics, Meylan, France). Apolipoprotein A-I (ApoA-I), ApoB, and lipoprotein (a) were assayed with an immunoturbidimetric method on an automated analyzer (Roche Diagnostics, France). LDL-cholesterol was calculated using the Friedewald formula, with very LDL-cholesterol (g/L) = triglycerides (g/L)/5, as long as triglyceride concentration was below 4 g/L [27]. Among metabolic markers, total or LDL- cholesterol were lower in CAD individuals, probably reflecting effects of lipid-lowering drugs in patients (Table 1).

**Table 1:** Clinical and biological characteristics of the study population

	Cases (n = 263)	Controls (n = 514)	*P* value
Age (years)	60.3 (8.0)	59.0 (8.3)	0.04
Education (years of schooling)	9.6 (3.0)	13.1 (4.3)	0.001 ^a^
Alcohol (g/day)	28.8 (28.5)	23.8 (24.1)	0.09 ^a^
Tobacco consumption number (pack/year)	41.5 (37.9)	17.2 (21.3)	0.001 ^a^
Physical activity (%)			0.001
No	27.4	18.5	
Medium	61.6	45.9	
High	11.0	35.6	
Waist (cm)	99.3 (10.7)	95.3 (9.9)	0.001
Body mass index (kg/m^2^)	27.5 (4.0)	26.9 (3.6)	0.04
Total cholesterol (g/L)	2.02 (0.38)	2.24 (0.38)	0.001
HDL-cholesterol (g/L)	0.43 (0.12)	0.55 (0.13)	0.001
LDL-cholesterol (g/L)	1.25 (0.34)	1.45 (0.32)	0.001
Triglyceride (g/L)	1.68 (0.89)	1.21 (0.77)	0.001 ^b^
ApoA-I (g/L)	1.23 (0.22)	1.50 (0.24)	0.001
ApoB (g/L)	1.04 (0.22)	1.08 (0.22)	0.03
Lipoprotein (a) (g/L)	0.47 (0.44)	0.30 (0.37)	0.001 ^b^
hs-CRP (mg/L)	17.2 (29.7)	3.1 (5.1)	0.001 ^a^
Gamma glutamyl transferase (IU/L)	62.8 (68.6)	45.2 (56.8)	0.001 ^a^
Fasting glucose (mmol/L)	5.93 (2.01)	5.43 (1.06)	0.19 ^a^
Serum insulin (UI/L)	15.9 (19.5)	10.0 (8.1)	0.001 ^a^
HOMA-IR	4.2 (4.8)	2.6 (2.8)	0.001 ^a^
Metabolic syndrome (NCEP ATP-III %)	48.5	17.7	0.001
Systolic blood pressure (mmHg)	137.0 (20.2)	137.4 (16.5)	0.82 ^a^
Resting heart rate (beat/mn)	63.7 (11.5)	62.8 (9.2)	0.79 ^a^
Ankle-arm index ≤0.9 (%)	33.6	1.6	0.001
Treatment for hypertension (%)	44.1	19.6	0.001
Treatment for diabetes (%)	23.2	5.2	0.001
Treatment for dyslipidemia (%)	63.5	23.3	0.001
s-RNY1-5p	10.42 (12.33)	1.32 (1.67)	0.001 ^a^
(RNY1-5p)^1/7^	1.33 (0.18)	0.95 (0.19)	0.001
Quartiles RNY1-5p (%)			0.001
Q1 : <0.47	0.4	37.9	
Q2 : 0.47–1.62	5.3	34.6	
Q3 : 1.61–4.70	28.5	23.4	
Q4 : >4.70	65.8	4.1	

Data are expressed as mean (±SD) or percentage (%)

^a^ Wilcoxon–Mann–Whitney test

^b^ Analyses performed on log transformed data

ApoA-I, Apolipoprotein A-I; ApoB, Apolipoprotein B; hs-CRP, High sensitive C-reactive protein; HOMA-IR, Homeostatic model assessment-insulin resistance; NCEP ATP-III, National Cholesterol Education Program’s Adult Treatment Panel III report

### Statistical analysis

Continuous variables were displayed as means and standard deviations. Categorical variables are presented as proportions. In univariate analyses, qualitative variables were compared with χ^2^ test (or Fisher exact test when necessary). Mean values of quantitative variables were compared by the Student *t*-test. The Shapiro–Wilks and the Levene tests were used to test the normality of distribution of residuals and the homogeneity of variances, respectively. When basic assumptions of the Student *t*-test were not satisfied, a logarithmic transformation of the variables was done or the Wilcoxon–Mann–Whitney test was performed. A seventh root transformation of s-RNY1-5p was made to approximate normal distribution of the variable (Additional file 5: Figure S4B and C). In cases and controls groups, associations of s-RNY1-5p with clinical characteristics and biological markers were tested using Spearman’s rank correlations.

Multivariate logistic regressions were carried out to test the statistical association of s-RNY1-5p levels with CAD. Analyses were performed firstly without adjustment and secondly after adjustment on classical cardiovascular risk factors, without any specific selection. The following cardiovascular risk factors were introduced in the model a priori: waist circumference, tobacco consumption (pack/year), total alcohol consumption, hs-CRP, apoA-I, apoB, hypertension (defined as systolic blood pressure >160 mmHg or diastolic blood pressure >95 mmHg or antihypertensive medication), diabetes (defined as blood glucose >7.8 mmol/L or anti-diabetic medication), statin drug, fibrate drug, and ankle-arm index, a clinical marker of peripheral arterial disease. Model assumptions were verified. Since the linearity of the relationship between s-RNY1-5p and the dependent variable (CAD status) was not verified, we performed analyses using quartiles of s-RNY1-5p. In these models, higher levels of s-RNY1-5p were strongly and significantly associated with CAD. The performance of these models was evaluated by analysis of the area under the receiver operating characteristic (ROC) curve (AUC). The internal validity of the prediction model was evaluated by bootstrapping [28] using 100 random samples (not shown). ROC curves were constructed, and models with and without s-RNY1-5p, were compared [29]. Analyses were two-tailed, and *P* <0.05 was considered to be significant.

### Ethics, permissions, and consent

Written informed consent was obtained from all participants involved in the study, including publication agreement. Biological collection was constituted according to the principles expressed in the Declaration of Helsinki and registered under number DC-2008-403 at the Ministry of Research and at the Regional Health Agency. The GENES study protocol was approved by the local Ethic Committee of the hospital of Toulouse (CHU Toulouse/INSERM, file 1-99-48, February 2000) and the national commission for data processing and freedoms (N° 900165).

Principles of laboratory animal care (NIH publication no. 85–23, revised 1985) were followed, as well those of the European Union guidelines on animal laboratory care. Animals were kept in a pathogen-free barrier facility and maintained in accordance with the Institutional Animal Care and Use Protocol of the University of Nice Sophia Antipolis and with appropriate National legislation concerning animal welfare. All procedures were approved by the Animal Care Committee of the Faculty of Medicine of the Nice Sophia Antipolis University (Comité Institutionnel d'Éthique Pour l'Animal de Laboratoire).

## Results and discussion

### Pro-apoptotic and atherogenic stimuli induce the expression of s-RNYs in macrophages

**Fig. 1:**
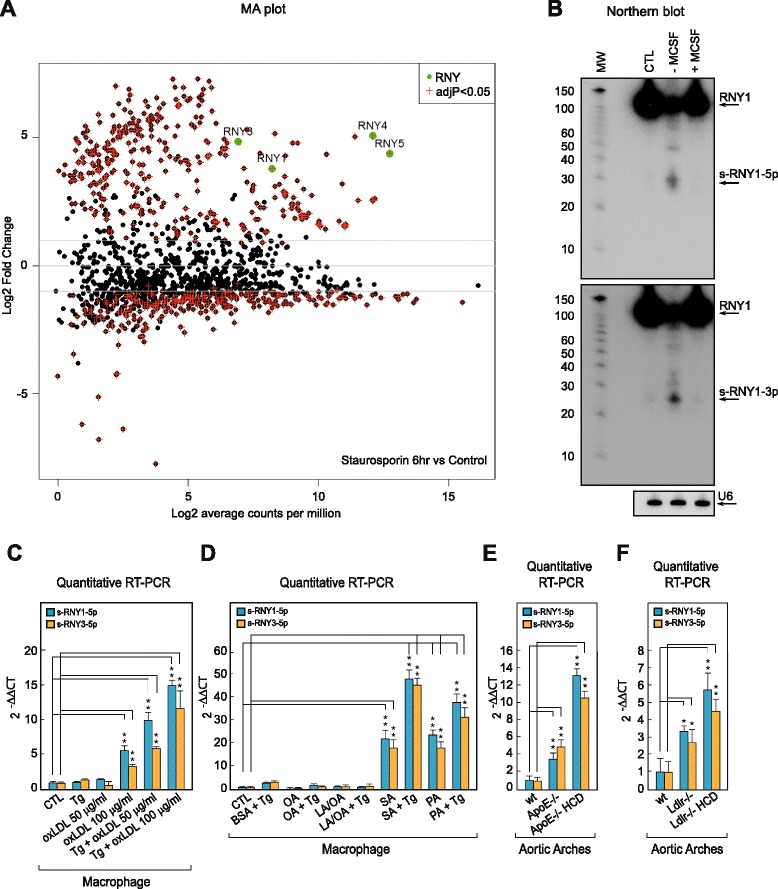
Apoptotic and atherogenic stimuli induce s-RNY expression in macrophages. **a** MA plot distribution from the high throughput sequencing analysis of differentially expressed small RNAs in human primary macrophages stimulated with 1 μM of staurosporine (STS) for 6 hours compared to control. Green dots indicated RNYs. **b** Northern blot detecting the indicated s-RNYs in apoptotic mouse bone marrow-derived macrophages (BMDMs) upon 36 h of M-CSF withdrawal treatment or reconstitution after 12 h of starvation. U6 snRNA was used as loading control. CTL indicates control. **c** RT-qPCR analysis of the indicated s-RNYs in mouse BMDMs incubated for 28 h with 0.25 μM of thapsigargin (Tg) alone or in combination with the indicated concentrations of oxidized LDL. The data were normalized by U2 snRNA and presented as mean and standard deviation (n per group = 8). **d** RT-qPCR analysis of the indicated s-RNYs in mouse BMDMs incubated for 18 h with 0.25 μM of Tg with bovine serum albumin (BSA), 0.25 mM of the indicated fatty acids in complex with BSA, or Tg plus the fatty acids. The data were normalized by U2 snRNA and presented as mean and standard deviation (n per group = 8). The unsaturated fatty acids were linoleic acid (LA) and oleic acid (OA), and the saturated fatty acids were stearic acid (SA) and palmitic acid (PA). RT-qPCR analysis of the indicated s-RNYs in control and *ApoE* ^−/−^ (**e**) or *Ldlr* ^*−/−*^ (**f**) aortic arches. The mice were fed with either chow diet or high cholesterol diet. The data were normalized by U2 snRNA and presented as mean and standard deviation (n per group = 8 for (**e**) and n per group = 5 for (**f**)). Student’s *t*-test: *P <0.05; **P <0.01

To discover novel small RNAs expressed in human and mouse apoptotic macrophages we performed a high-throughput small RNA sequencing in primary macrophages stimulated with either STS or M-CSF withdrawal (Additional file 6: Tables S1 and Additional file 7: Table S2). We observed that RNY-derived small RNAs were among the most upregulated and expressed small RNAs detected in human apoptotic macrophages (Fig. 1a, Additional file 8: Figure S1 and Additional file 9: Figure S2A, and Additional file 10: Table S3). This result was also confirmed in mouse apoptotic macrophages (Additional file 9: Figure S2B and Additional file 10: Table S3), indicating that RNY processing into small species is evolutionarily conserved. We found that the RNY-derived small RNAs vary in length from ~24 to ~34 nt and map at the end of the stem regions of RNYs (Additional file 8: Figures S1, Additional file 9: Figure S2B and C). We refer to these small RNAs as s-RNYs. RNY genes account for four copies in human (RNY1, 3, 4, and 5) and two in mouse genomes (RNY 1 and 3), and their sequence is conserved in vertebrates [30]. RNYs are characterized by extensive base-pairing of the 5′ and 3′ regions and by the association with Ro60 and La/SSB [30]. Interestingly, only in human macrophages we detected many reads mapped on RNY pseudogenes. Indeed, in the human genome, the presence of 966 pseudogenes derived from the RNYs has been previously reported, whereas they can be rarely found in mouse [31]. Therefore, the expression of different types of RNY fragments in human apoptotic macrophages reflects the much bigger copy number of RNY genes and pseudogenes in human. Accordingly, detection of fragments derived from the so-called RNY pseudogenes was previously observed in human serum and plasma [32], thus suggesting that they should be considered as belonging to a multicopy gene family rather than pseudogenes. Northern blot, quantitative RT-PCR (RT-qPCR), and high-throughput RNA sequencing from 25–40 nt size selected RNA purified by polyacrylamide gel electrophoresis confirmed that the expression of s-RNYs was induced in primary macrophages treated with different apoptotic stimuli (Fig. 1b, Additional file 2: Figure S3A, B, and C, Additional file 11: Table S4).

Although the mouse s-RNY1-5p have been previously classified as piRNA (piRNABank, [33]) and the human s-RNY5-3p as microRNA [34], nowadays s-RNYs are considered as belonging to an independent class of small RNAs. Indeed, while s-RNYs are associated with Ro60 (Additional file 2: Figures S3D) [35] to form a ribonucleoprotein complex of unknown function, miRNAs and piRNAs associate with Argonaute 2 or Piwi, respectively, to silence target RNAs [36].

**Fig. 2:**
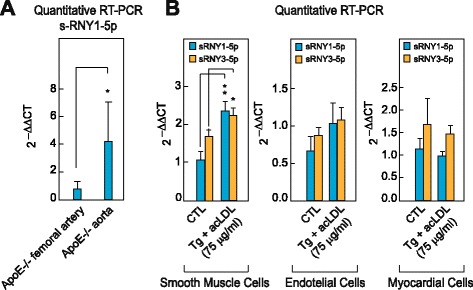
s-RNY expression is induced in atherosclerotic areas and in smooth muscle foam cells. **a** RT-qPCR analysis of s-RNY1-5p in *ApoE* ^−/−^ aortic arches and femoral arteries. The data were normalized by U2 snRNA and are presented as mean and standard deviation (n per group = 5). **b** RT-qPCR analysis of the indicated s-RNYs in CRL 2797 cells (smooth muscle cells; left panel, n per group = 6), CRL 2181 cells (endothelial cells; central panel, n per group = 7), primary myocardial cells (right panel, n per group = 3) incubated for 28 h with 0.25 μM thapsigargin in combination with the indicated concentration of acetylated LDL and control. The data were normalized by U2 snRNA and are presented as mean and standard deviation. Student’s *t*-test: *P <0.05, **P <0.01

A number of lipids and lipoproteins associated with atherosclerotic diseases conspires with ER stress to cause macrophage apoptosis and progression of atherosclerotic lesions [4]. To determine whether s-RNYs are induced in macrophages stimulated with athero-relevant stimuli, we treated BMDMs with oxLDL, alone or in presence of the ER stessor Tg. Whereas low dose of oxLDL or Tg alone had no effect, higher levels of oxLDL or both reagents together synergistically induced s-RNY expression (Fig. 1c and Additional file 2: Figure S3E). Similar results were observed with acLDL (Additional file 2: Figure S3F). We then tested whether different types of fatty acids could also trigger s-RNY induction in macrophages. Only saturated fatty acids, such as palmitic acid and stearic acids, induced s-RNY expression and this effect was increased markedly in presence of Tg (Fig. 1d and Additional file 2: Figure S3G). Moreover, to test whether the s-RNYs are induced in animal models for atherosclerosis, we compared *ApoE*^−/−^, *Ldlr*^*−/−*^, and control mice. As shown in Fig. 1e,f, RT-qPCR from microdissected aortic arches revealed that s-RNYs were significantly upregulated in *ApoE*^−/−^ and *Ldlr*^*−/−*^ mice compared to control, and that the HCD significantly increased their expression. However, s-RNYs were not modulated in atheroprotected sites, such as femoral artery (Fig. 2a), suggesting specificity for atherosclerotic lesions. To assess the cellular origin, we measured the levels of s-RNY expression in different cell types involved in cardiovascular disease. Besides macrophages, we investigated endothelial cells, primary cardiomyocytes, and SMCs. Among those cells, only SMCs showed a significant increase of s-RNYs upon acLDL and Tg stimulus (Fig. 2b).

**Fig. 3:**
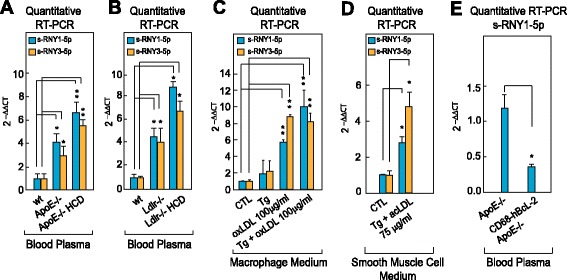
s-RNYs are released in the extracellular milieu by foam cells and in the peripheral blood of two mouse models for atherosclerosis. RT-qPCR of the indicated s-RNYs in the blood plasma of *ApoE* ^−/−^ (**a**) or *Ldlr* ^−/−^ (**b**) mice or control (*wt*). Mice were fed with either chow diet or high cholesterol diet. Data were normalized by using cel-miR-39 and are presented as mean and standard deviation (n per group = 8 for (**a**) and n per group = 5 for (**b**)). **c** RT-qPCR analysis of the indicated s-RNYs from the medium of bone marrow-derived macrophages (n per group = 4). **d** RT-qPCR analysis of the indicated s-RNYs from the medium of CRL 2797 cells (smooth muscle cells) incubated for 28 h with 0.25 μM thapsigargin in combination with the indicated concentration of acetylated LDL and control. The data were normalized by cel-miR-39 and are presented as mean and standard deviation (n per group = 4). **e** RT-qPCR of s-RNY1-5p in the blood plasma of *CD68-hBcl-2 ApoE* ^−/−^ or control (*ApoE* ^−/−^). Data were normalized by cel-miR-39 and are presented as mean and standard deviation (n per group = 4). Student’s *t*-test: *P <0.05, **P <0.01

Because s-RNYs have been found in significant amounts in the blood [37], we compared s-RNY expression levels in the blood of *ApoE*^−/−^, *Ldlr*^*−/−*^ and control mice. As shown in Fig. 3a,b, RT-qPCR from blood plasma revealed that s-RNYs were significantly upregulated in *ApoE*^−/−^ and *Ldlr*^*−/−*^ mice compared to control, and even more when mice were fed with a HCD. Consistently with the results on cultured cells, a significant upregulation of s-RNYs was observed in the medium of both lipid-laden macrophages and SMCs (Fig. 3c,d). Importantly, two main types of foam cells were described in atherosclerotic lesions: those derived from macrophages and those derived from SMCs [38]. Unfortunately, the contribution of these two cell types to the total lesional foam cell population in atherosclerosis has not been elucidated yet, mainly because lipid-laden SMCs assume a macrophage-like phenotype [38]. Therefore, to determine the role of macrophages as a source of serum s-RNYs, we used the *ApoE*^*−/−*^ mouse model in which the human isoform of the anti-apoptotic Bcl-2 gene was expressed only in macrophages under the CD68 promoter [5]. This mouse model, here called *CD68-hBcl-2 ApoE*^*−/−*^, is characterized by the dramatic downregulation of the apoptosis in macrophages, ultimately resulting in a significant reduction of the size of atherosclerotic advanced lesions [5]. Importantly, our results indicate that circulating s-RNY expression was significantly downregulated in *CD68-hBcl-2 ApoE*^*−/−*^ mice compared to the control *ApoE*^*−/−*^ mice (Fig. 3e). Altogether, these data identified apoptotic macrophage-derived foam cells as the main source of s-RNYs in atherosclerosis.

### s-RNYs are upregulated in the serum of patients with coronary artery disease (CAD)

**Fig. 4:**
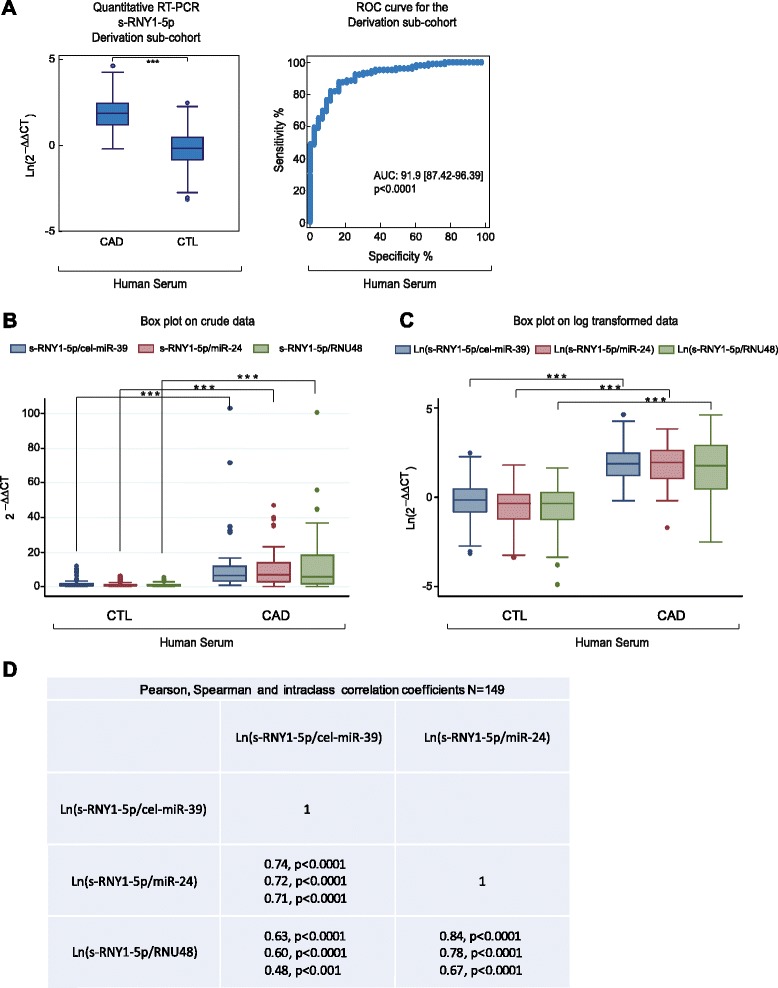
Circulating s-RNY1-5p expression is induced in patients with CAD. **a** Box plot showing the expression in natural logarithmic scale of circulating s-RNY1-5p in the derivation cohort composed by the serum of 43 coronary artery disease (CAD) patients versus 106 control individuals (left panel). Data derived from RT-qPCR, normalized using cel-miR-39, and presented as mean and standard deviation. Receiver operating characteristic (ROC) curve for predicting CAD with s-RNY1-5p was based on the RT-qPCR data of n = 149 (right panel). The area under the ROC curve is indicated. **b**, **c** Box plots showing, in the derivation cohort (43 CAD patients and 106 control individuals), the expression of circulating s-RNY1-5p normalized with the indicated reference genes, in linear scale (**b**) and natural logarithmic scale (**c**). **d** Pearson, Spearman, and interclass correlation coefficients among the circulating s-RNY1-5p values normalized with the indicated reference genes. Student’s *t*-test: ***P <0.001

Because changes in the level of circulating s-RNY expression were observed in different pathophysiological processes, such as some types of cancer [37, 39], it is conceivable that expression of circulating s-RNYs may also be affected in atherosclerotic cardiovascular diseases. To test this hypothesis, we measured the level of serum s-RNY1-5p in the GENES study cohort [24], including 43 male patients with stable CAD (cases) and 106 age-matched healthy men (derivation sub-cohort). Average levels of circulating s-RNY1-5p, normalized with the exogenous spike-in cel-miR-39, were significantly lower in controls than in CAD patients (*P* <0.001; Fig. 4a). The same results were observed upon normalization with two endogenous small RNAs, namely the miR-24 [21] or the RNU48 [22] (Fig. 4b,c), indicating that our data were independent on the type of normalizer used (Fig. 4d). We then validated this first observation in an independent sub-cohort, including 220 CAD patients and 408 controls selected from the GENES cohort (validation sub-cohort; Additional file 5: Figure S4A).

**Fig. 5:**
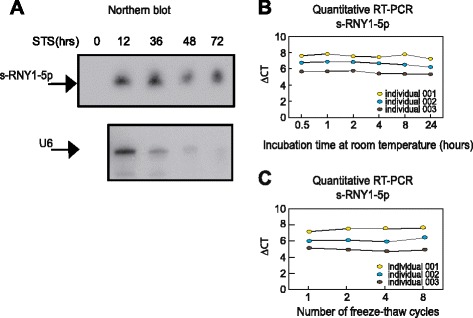
s-RNY1-5p is stable in extracellular environment. **a** Northern blot analysis showing the stability of s-RNY1-5p and U6 snRNA in the medium of bone marrow-derived macrophages stimulated with 1 μM of staurosporine at the indicated time points. Total RNA was isolated from the medium. **b**, **c** RT-qPCR analysis from three coronary artery disease patients showing the stability of s-RNY1-5p in the serum in either prolonged room temperature incubation (**b**) or different cycles of freeze-thaw as indicated (**c**). Data were normalized by using the synthetic non-mammalian cel-miR-39, which was added to the samples before the RNA extraction. Each ΔCt (corresponding to Ct^s-RNY1-5p^-Ct^cel-miR-39^) is the average of three technical replicates

**Fig. 6:**
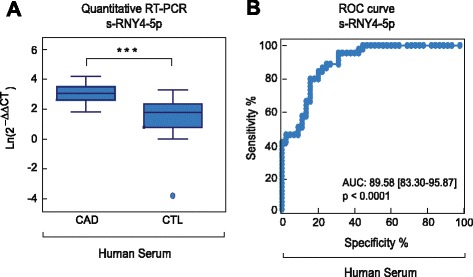
Circulating s-RNY4-5p expression is induced in patients with coronary artery disease (CAD). **a** Box plot showing the expression in natural logarithmic scale of circulating s-RNY4-5p in the serum of CAD patients (n = 45) versus control individuals (CTL, n = 45). Data derived from RT-qPCR were normalized using cel-miR-39, and presented as mean and standard deviation. **b** Receiver operating characteristic (ROC) curve for predicting CAD with s-RNY4-5p. The ROC curve was constructed using s-RNY4-5p based on the RT-qPCR data (n = 90). The area under the ROC curve is given and 95 % confidence interval was indicated in square brackets. Student’s *t*-test: ***P <0.001

Like microRNAs, s-RNYs are also strikingly stable in cell culture medium compared to U6 snRNA (Fig. 5a), and in human serum from CAD patients after incubation at room temperature for up to 24 h (Fig. 5b) or up to eight cycles of freeze-thaw (Fig. 5c). Extracellular microRNA stability is caused by their association with protein complexes, microvesicles, exosomes, apoptotic bodies, or lipoproteins [40]. Plasma s-RNYs have been found circulating as part of a protein complex with a mass between 100 and 300 kDa [32]. Similarly, serum s-RNY4-5p was upregulated in 45 CAD patients compared to 45 controls from the same study cohort (Fig. 6a,b), whereas s-RNY3-5p was undetectable. Therefore, these data indicate that circulating s-RNYs are induced in CAD patients and may be considered as novel biomarkers for CAD status.

**Fig. 7:**
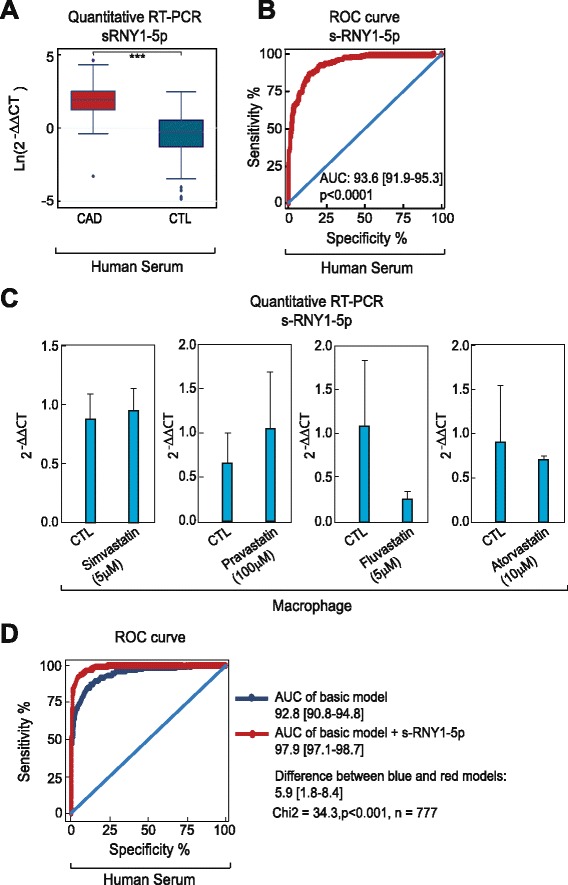
s-RNY1-5p is an independent diagnostic biomarker for coronary artery disease (CAD). **a** Box plots showing the expression in natural logarithmic scale of circulating s-RNY1-5p in the serum of CAD patients (n = 263) versus control individuals (CTL, n = 514). Data derived from RT-qPCR were normalized using cel-miR-39, and presented as mean and standard deviation. **b** Receiver Operating Characteristic (ROC) curve for predicting CAD with s-RNY1-5p. The ROC curve was constructed using s-RNY1-5p based on the RT-qPCR data (n = 777). The area under the ROC curve is given and 95 % confidence interval was indicated in square brackets. The diagonal line indicates a test with an area under the ROC curve of 50 %; *P* <0.0001. **c** RT-qPCR analysis of the indicated s-RNYs in bone marrow-derived macrophages incubated for 24 h with the indicated concentrations of different statins. The data were normalized by U2 snRNA and presented as mean and standard deviation (n per group = 3). **d** ROC, in blue the curve for classical risk factors and markers (hypertension, dyslipidemia, diabetes, smoking, waist circumference, ankle-arm index, ApoA-I, and hs-CRP), in red the same classical risk factors and markers with s-RNY1-5p. The ROC curve was constructed using s-RNY1-5p expression based on the RT-qPCR data (n = 777). The area under the ROC curve is given and 95 % confidence interval was indicated in square brackets. Student’s *t*-test: ***P <0.001

We then compared s-RNY1-5p expression levels with cardiovascular risk factors or markers within the cohort study to assess its value as a novel biomarker. In Table 1, we report metabolic and clinical variables, including serum sRNY1-5p levels, of the whole case–control cohort (derivation and validation sub-cohorts pulled together, *P* <0.001; Fig. 7a,b). Briefly, increased prevalence of classical risk factors was recorded in CAD patients: hypertension, diabetes, dyslipidemia (defined as total cholesterol >2.50 g/L), and smoking habits. BMI and waist circumference were higher in CAD-affected than in control individuals, while educational level and physical activity were lower. Averages of alcohol consumption were not significantly different between cases and controls. However, alcohol patterns were different, with a higher percentage of moderate alcohol consumption (<40 g/day) in controls and, conversely, a higher percentage of both abstinent and excessive drinkers (≥40 g/day) in cases. Among metabolic markers, total cholesterol or LDL-cholesterol were lower in CAD individuals, reflecting effects of lipid-lowering drugs in patients. However, CAD individuals displayed hyperglycemia, higher levels of triglycerides and lipoprotein (a), increased CRP, and lower HDL markers. Altogether, 48.5 % of CAD individuals had features of metabolic syndrome, according to the National Cholesterol Education Program’s Adult Treatment Panel III report definition, versus 17.7 % of control subjects. Transformation of s-RNY1-5p levels in the whole case–control cohort into its exp^1/7^ derivative yielded normal distribution and was further used for statistics (Additional file 5: Figure S4B and C). Spearman rank correlation coefficients between the levels of serum s-RNY1-5p and metabolic parameters or cardiovascular risk markers were calculated (Table 2). In CAD patients (n = 263) and control individuals (n = 514), serum s-RNY1-5p was positively correlated to the pro-atherogenic apoB-containing lipoproteins and with an inflammatory condition, as documented by an elevated hs-CRP. In controls, negative correlations were found with the level of atheroprotective apoA-I and with alcohol consumption, whereas a positive correlation was found with atherogenic LDL-cholesterol. Age, glycemic parameters, and some environmental factors, such as physical activity and school duration, were not associated with s-RNY1-5p. No statistical interaction could be found between, on the one hand, the coronary status and, on the other hand, hs-CRP and apoB levels regarding s-RNY1-5p levels (Table 3), indicating that the association of s-RNY1-5p with CAD status was independent of the concentration of those two markers. The independency between the expression levels of serum s-RNY1-5p and hs-CRP was also validated in a group of 17 male individuals with blood infection originated from different entry portals: lung, bone, cutaneous, digestive, and urinary track (Additional file 4: Table S5). Comparisons were made with 35 age-matched controls from the GENES study. Although, both sRNY1-5p (*P* <0.02) and hs-CRP (*P* <0.001) levels were elevated in infected patients, sRNY1-5p induction was moderate compared to that found in CAD patients, conversely, hs-CRP levels were far higher in infected subjects. Moreover, no significant correlation was found between both markers (Spearman rank correlation coefficient: 0.15, *P* = 0.56). Hence, we concluded that, whereas CRP is an unambiguous marker for systemic inflammation, sRNY1-5p might be more specifically retaliated to the local inflammatory process occurring in atherosclerotic lesions.

**Table 2:** Relationships between s-RNY1-5p and classical cardiovascular risk factors in controls and cases

	Controls (n = 514)	Cases (n = 263)
Age (years)	−0.07 (0.10)	−0.09 (0.14)
School (years of schooling)	0.01 (0.88)	−0.05 (0.39)
Alcohol (g/day)	−0.12 (0.008)	−0.04 (0.50)
Tobacco consumption number (pack/year)	0.04 (0.50)	0.14 (0.03)
Levels of physical activity	−0.01 (0.88)	−0.04 (0.50)
Waist (cm)	−0.09 (0.04)	−0.02 (0.80)
Body mass index (kg/m^2^)	−0.07 (0.14)	−0.03 (0.63)
Total cholesterol (g/L)	0.07 (0.11)	0.11 (0.09)
HDL-cholesterol (g/L)	−0.03 (0.53)	−0.02 (0.82)
LDL-cholesterol (g/L)	0.09 (0.05)	0.12 (0.06)
Triglyceride (g/L)	0.03 (0.52)	0.01 (0.90)
ApoA-I (g/L)	−0.09 (0.04)	−0.08 (0.17)
ApoB (g/L)	0.18 (<0.001)	0.18 (0.003)
Lipoprotein (a) (g/L)	0.04 (0.39)	0.07 (0.24)
hs-CRP (mg/L)	0.16 (<0.001)	0.14 (0.03)
Gamma glutamyl transferase (IU/L)	0.04 (0.41)	0.11 (0.09)
Fasting glucose (mmol/L)	0.06 (0.22)	−0.04 (0.54)
Serum insulin (UI/L)	−0.07 (0.14)	−0.02 (0.81)
HOMA-IR	−0.05 (0.24)	−0.05 (0.46)
Systolic blood pressure (mmHg)	−0.01 (0.74)	−0.08 (0.19)
Resting heart rate (beat/mn)	0.04 (0.42)	0.03 (0.64)

Spearman rank correlation coefficients (*P* value)

ApoA-I, Apolipoprotein A-I; ApoB, Apolipoprotein B; hs-CRP, High sensitive C-reactive protein; HOMA-IR, Homeostatic model assessment-insulin resistance

**Table 3:** s-RNY1-5p level according to high sensitive C-reactive protein (hs-CRP) levels, apolipoprotein B (ApoB) levels, statin therapy, and fibrate therapy and case–control (CC) status

CRP (mg/L)	CC	(s-RNY1-5p)^1/7^	CC effect	hs-CRP effect	CC × hs-CRP interaction
<6	Case (n = 121)	1.30 (0.17)	0.001	0.03	0.72
≥6	Case (n = 142)	1.34 (0.18)			
<6	Control (n = 449)	0.95 (0.18)			
≥6	Control (n = 65)	0.98 (0.20)			
ApoB (g/L)	CC	(s-RNY1-5p)^1/7^	CC effect	ApoB effect	CC × ApoB interaction
<1.02	Case (n = 115)	1.30 (0.18)	0.001	0.001	0.79
≥1.02	Case (n = 148)	1.35 (0.17)			
<1.02	Control (n = 200)	0.92 (0.19)			
≥1.02	Control (n = 314)	0.97 (0.19)			
Statin therapy	CC	(s-RNY1-5p)^1/7^	CC effect	statin effect	CC × statin interaction
No	Case (n = 115)	1.34 (0.18)	0.001	0.15	0.78
Yes	Case (n = 148)	1.31 (0.18)			
No	Control (n = 428)	0.95 (0.19)			
Yes	Control (n = 86)	0.93 (0.20)			
Fibrate therapy	CC	(s-RNY1-5p)^1/7^	CC effect	fibrate effect	CC × fibrate interaction
No	Case (n = 242)	1.33 (0.17)	0.001	0.21	0.46
Yes	Case (n = 21)	1.28 (0.19)			
No	Control (n = 472)	0.95 (0.19)			
Yes	Control (n = 42)	0.94 (0.18)			

The association of s-RNY1-5p with CAD was also independent of lipid-lowering treatments (statins and fibrates, Table 3). We further investigated the effects of four different statins, namely fluvastatin, simvastatin, lovastatin, and pravastatin, on the s-RNY expression in macrophages. As shown in Fig. 7c, none of the statins used significantly impacted on s-RNY expression in BMDMs, confirming that changes on serum s-RNY expression are independent from treatments with statins.

To further explore the association with CAD, a ROC curve was constructed using s-RNY1-5p (Fig. 7b, AUC = 93.6 [91.9–95.3]) together with classical cardiovascular risk factors, including hypertension, dyslipidemia, diabetes, smoking, waist circumference, ankle-arm index, ApoA-I, and hs-CRP (basic model in Fig. 7d, AUC = 92.8 [90.8–94.8]). As shown in Fig. 7d, the resultant ROC curve displays a greater AUC compared to the basic model (AUC = 97.9 [97.1–98.7], *P* <0.001 for the difference). Moreover, quartile ranking of s-RNY1-5p was performed and the odds ratios with 95 % confidence intervals were determined (Table 4). In these models, higher levels of s-RNY1-5p were strongly and significantly associated with CAD. In multivariate analyses, this association was maintained when adjustments were performed on cardiovascular risk factors, markers, or factors associated with s-RNY1-5p, including waist circumference, tobacco consumption (pack/year), ankle-arm index, hypertension, diabetes, hs-CRP, apoA-I, apoB, alcohol consumption, and lipid-lowering treatments.

**Table 4:** Association of s-RNY1-5p with coronary artery disease (case–control study)

Models with	Q1 + Q2	Q3	Q4
	OR (95 % CI)	OR (95 % CI)	OR (95 % CI)
s-RNY1-5p	1	15.4 (8.5–27.8)	204.7 (102.3–409.6)
s-RNY1-5p ^a^	1	18.0 (7.9–40.8)	206.7 (76.2–560.5)

Quartiles for s-RNY1-5p: 0.47, 1.62, 4.70

Quartiles Q1 and Q2 were grouped together since there was only one case in Q1 (patients)

^a^ Adjusted for waist circumference, tobacco consumption (pack/year), ankle-arm index, hypertension, diabetes, hs-CRP (log), ApoA-I, ApoB, total alcohol consumption, statin drug and fibrate drug

OR, Odds ratio; 95 % CI, 95 % Confidence intervals

Altogether, these results evidence a strong positive independent association between the levels of serum s-RNYs and CAD risk, overall supporting the hypothesis that s-RNY1-5p is an independent signature of CAD, which can improve CAD diagnosis and risk stratification.

## Conclusions

In conclusion, the present study demonstrates the generation of RNY-derived small RNAs, namely s-RNYs, in macrophages incubated with pro-apototic and atherogenic stimuli. Significantly increased levels of s-RNYs are observed in the blood of mouse models for atherosclerosis and CAD patients, in which they are independently associated with CAD risk factors and markers.

In the future, s-RNYs could be used as independent diagnostic biomarkers for CAD to better evaluate cardiovascular risk, when combined into a “multimarker” score [41]. Furthermore, measurement of circulating s-RNY levels would be a valuable companion diagnostic to monitor foam cell apoptosis during atherosclerosis pathogenesis and to evaluate a patient’s responsiveness to future therapeutic strategies aiming to attenuate apoptosis in foam cells in advanced atherosclerotic lesions.

Many publications previously showed that other non-coding RNAs, mostly miRNAs, can be potentially used as biomarkers of cardiovascular disorders [42]. miRNAs and s-RNYs share the same advantages, including high sensitivity and specificity detection, the fact that a single sample can be used for the detection of many small RNAs allowing the determination of specific signatures, very high stability, and serum abundance [43]. However, our data indicate that s-RNY1-5p correlates much more with a CAD status compared to other CAD-specific miRNAs, including miR-17, miR-92a, miR-126, miR-133, miR-145, miR-155, and miR-208 (Table 5), indicating the potential biomedical implications of s-RNY measurement as a diagnostic tool. Future epidemiological studies on perspective cohorts will provide useful information on prognostic values of serum s-RNY expression. In short, our work represents a step forward toward a critical assessment of circulating small non-coding RNAs (but not only miRNAs) as biomarkers for cardiovascular diseases.

**Table 5:** Comparative analysis between s-RNY1-5p and selected coronary artery disease-associated miRNAs in the derivation cohort

Normalization by cel miR-39	2^-ΔΔCt^ in CAD	2^-ΔΔCt^ in CTL	ROC Area	*P* value	Std. Error	95 % Confidence interval
sRNY1-5p	12.15861132	1.506803056	0.919	<0.0001	0.02286	0.8742–0.9639
miR17	0.804077414	0.920930566	0.5687	0.1898	0.0501	0.4705–0.6669
miR92a	4.91098005	0.976527138	0.5919	0.07926	0.05539	0.4833–0.7005
miR126	2.592316951	1.265612624	0.6784	0.0006643	0.04854	0.5832–0.7735
miR133	4.765530301	1.095526208	0.5965	0.06534	0.05355	0.4915–0.7015
miR145	4.239215843	0.979840396	0.5083	0.8735	0.05351	0.4034–0.6132
miR155	8.677582108	1.039014581	0.6255	0.01659	0.05367	0.5203–0.7307
miR208a	5.554050756	1.058798935	0.7246	<0.0001	0.0484	0.6297–0.8195
Normalization by RNU48	2^-ΔΔCt^ in CAD	2^-ΔΔCt^ in CTL	ROC Area	*P* value	Std. Error	95 % Confidence interval
sRNY1-5p	13.95354052	1.069518668	0.8341	<0.0001	0.0442	0.7475–0.9208
miR17	1.084053274	0.949856557	0.5428	0.414	0.05115	0.4425–0.6431
miR92a	1.20101817	1.033002128	0.7865	<0.0001	0.03945	0.7092–0.8639
miR126	1.44021571	1.072261942	0.509	0.8636	0.06102	0.3894–0.6286
miR133	1.334960926	1.126558473	0.5608	0.2459	0.05328	0.4563–0.6652
miR145	1.52987889	0.939525323	0.749	<0.0001	0.04181	0.6670–0.8310
miR155	0.891246591	1.064118053	0.527	0.6064	0.05249	0.4241–0.6299
miR208a	0.247755721	1.009457364	0.6772	0.0007879	0.04678	0.5855–0.7689
Normalization by miR-24	2^-ΔΔCt^ in CAD	2^-ΔΔCt^ in CTL	ROC Area	*P* value	Std. Error	95 % Confidence interval
sRNY1-5p	11.26524945	0.975729203	0.9382	<0.0001	0.0236	0.8919–0.9845
miR17	1.509090429	0.934815204	0.6605	0.002226	0.04591	0.04591
miR92a	2.551324286	0.952726947	0.767	<0.0001	0.04154	0.6856–0.8484
miR126	1.754084858	1.200874169	0.5595	0.2568	0.05284	0.4559–0.6631
miR133	2.155706321	0.928731186	0.5996	0.05769	0.05292	0.4958–0.7033
miR145	3.099747057	1.041255052	0.7506	<0.0001	0.04349	0.6653–0.8359
miR155	1.828043362	1.008436566	0.5143	0.7853	0.05613	0.4043–0.6243
miR208a	1.334878917	1.120233854	0.6398	0.008176	0.0532	0.5355–0.7441

CAD, Coronary artery disease; CTL, Control; ROC, Receiver operating curve

### Accession numbers

Experimental datasets have been deposited in the NCBI Gene Expression Omnibus (GEO) [44] under the Series record numbers: GSE63802. Link to the direct access: [45].

## Additional files

Additional file 1: Table S6. Oligonucleotides used for RT-PCR, cloning and Northern blot analyses. (PDF 19 kb) Additional file 2: Figure S3. s-RNY expression in apoptotic and lipid-laden macrophages. (A) Northern blot analysis detecting the indicated s-RNYs in human primary macrophages stimulated with 1 μM of staurosporin at the indicated time points. U6 snRNA was used as loading control. (B) Northern blot analysis detecting the indicated s-RNY in bone marrow-derived macrophages (BMDMs) after macrophage-colony stimulating factor withdrawal and reconstitution. (C) Endoplasmic reticulum stress renders macrophages susceptible to apoptosis in the face of other pro-apoptotic stimuli [46]. Quantitative RT-PCR analysis of the indicated s-RNYs in BMDMs incubated for 18 h with 10 mg/mL lipoteichoic acid alone or in combination with 0.25 μM thapsigargin (Tg). The data were normalized by U2 snRNA (n per group = 4). (D) RNA immunoprecipitation analysis of either Ro60 or Argonaute 2 and s-RNY1-5p in BMDMs. RNA was isolated from immunoprecipitation and analyzed by RT-qPCR. BMDMs were left unstimulated or stimulated with 100 μg/mL of oxidized LDL (oxLDL) and 0.25 μM Tg for 28 h. Data are presented as mean and standard deviation (n per group = 3). (E) Northern blot analysis detecting s-RNY1-3p in BMDMs stimulated for 28 h with the indicated concentration of oxLDL in combination with 0.25 μM Tg. U6 snRNA was used as loading control. (F) RT-qPCR analysis of the indicated s-RNYs in BMDMs incubated for 28 h with 0.25 μM Tg in combination with the indicated concentration of acetylated-LDL (acLDL) and control (CTL). The data were normalized by U2 snRNA (n per group = 4). (G) Northern blot analysis using the indicated probes recognizing different parts of RNY1 in BMDMs stimulated with 0.25 mM of palmitic acid for 18 h. U6 snRNA was used as loading control. Student’s *t*-test: ***P* <0.01. (PDF 202 kb) Additional file 3: Figure S5. Control experiments to assess the specificity of the RT-qPCR for s-RNYs. (A) RT-qPCR analysis detecting the indicated s-RNYs in bone marrow-derived macrophages (BMDMs) transfected with 2′-O-Me-RNA antisense oligonucleotides to s-RNYs or control. BMDMs were left unstimulated or stimulated with 0.25 mM of palmitic acid and 0.25 μM thapsigargin for 18 h, and total RNA was isolated and analyzed. Data are presented as mean and standard deviation (n per group = 3), and normalized with the input for the RT-qPCR. (B) Snapshot sequence read from Chromas Lite 2.1 (Technelysium Pty Ltd) showing the RT-PCR product corresponding to s-RNY1-5p from patients with coronary artery disease. Student’s *t*-test: ***P* <0.01. (PDF 89 kb) Additional file 4: Table S5. Levels of high sensitive C-reactive protein and s-RNY1-5p in infected and non-infected individuals. Portal of entries and pathogens were identified. (XLS 38 kb) Additional file 5: Figure S4. Validation experiment to assess the specificity of the s-RNY1-5p upregulation in serum of coronary artery disease (CAD) patients compared to healthy control individuals. (A) Box plot showing the expression in natural logarithmic scale of circulating s-RNY1-5p in the validation cohort of 220 CAD patients and 408 controls (left panel). Data derived from RT-qPCR, normalized using cel-miR-39, and presented as mean and standard deviation. Receiver operating characteristic (ROC) curve for predicting CAD with s-RNY1-5p was based on the RT-qPCR data of n = 628 (right panel). The area under the ROC curve for each cohort is indicated. Student’s *t*-test: ****P* <0.0001. (B) Density histogram of s-RNY1-5p levels in the serum of 777 individuals (263 CAD cases and 514 controls) from the GENES study, expressed in crude four values (upper histogram) and values transformed into seventh root (lower histogram). (C) Box plots showing the transformed expression of circulating s-RNY1-5p in the serum of coronary patients (CAD, n = 263) versus healthy control (CTL, n = 514). Data derived from RT-qPCR, normalized using cel-miR-39, and presented as mean and standard deviation. (PDF 387 kb) Additional file 6: Tables S1. Bioinformatic analysis of the high-throughput small RNAs sequencing of human primary macrophages stimulated with staurosporine at the indicated time (highlighted in pink the RNY-derived small RNAs). (XLS 1622 kb) Additional file 7: Table S2. Bioinformatic analysis of the high-throughput small RNAs sequencing of mouse bone marrow-derived macrophages stimulated with macrophage-colony stimulating factor withdrawal (highlighted in pink the RNY-derived small RNAs). (XLS 1017 kb) Additional file 8: Figure S1. s-RNY induction in apoptotic human primary macrophages. Bioinformatic analysis of the high-throughput small RNAs sequencing showing the upregulation of small RNA reads derived from RNYs (s-RNYs) in human primary macrophages stimulated with 1 μM of staurosporine at the indicated time points. University of California, Santa Cruz (UCSC) genome browser snapshot encompassing an ~0.2 kb segment of DNA spanning the *RNY* loci. (PDF 979 kb) Additional file 9: Figure S2. s-RNY induction in apoptotic mouse primary macrophages. (A) MA plot distribution of differentially expressed small RNAs in human primary macrophages stimulated with 1 μM of staurosporine (STS) for 4 hours compared to control. Green dots indicated RNYs. (B) Bioinformatic analysis of the high-throughput small RNAs sequencing showing the upregulation of small RNA reads derived from RNYs (s-RNYs) in bone marrow-derived macrophages after macrophage-colony stimulating factor withdrawal. University of California, Santa Cruz (UCSC) genome browser snapshot encompassing an ~0.2 kb segment of DNA spanning the *RNY* loci. (C) Bioinformatic analysis of small RNA-Seq data sets identifying a consensus sequence of s-RNYs in human primary macrophages after STS treatment. The bar graph on the top shows the localization of s-RNYs in RNYs. (PDF 657 kb) Additional file 10: Table S3. Statistical analysis of the small RNAs expression in human primary macrophages stimulated with staurosporine at the indicated time and in mouse bone marrow-derived macrophages stimulated with macrophage-colony stimulating factor withdrawal and controls. (XLS 911 kb) Additional file 11: Table S4. Bioinformatic analysis of the high-throughput small RNAs sequencing of 25–40 nt size selected RNAs from bone marrow-derived macrophages stimulated with macrophage-colony stimulating factor withdrawal (highlighted in pink the RNY-derived small RNAs). (XLS 439 kb)

## Abbreviations

acLDL: Acetylated LDL
AUC: Area under the receiver operating characteristic curve
BMDMs: Bone marrow-derived macrophages
CAD: Coronary artery disease
ER: Endoplasmic reticulum
HCD: High cholesterol diet
hs-CRP: High sensitive C-reactive protein
M-CSF: Macrophage-colony stimulating factor
nt: nucleotides
oxLDL: oxidized LDL
RT-qPCR: Quantitative RT-PCR
ROC: Receiver operating characteristic
SMC: Smooth muscle cell
s-RNYs: small-RNYs
STS: Staurosporin
Tg: Thapsigargin
