# Supplementary material for: RNY-derived small RNAs as a signature of coronary artery disease
Source: BMC Med. 2015 Oct 8;13:259. doi: 10.1186/s12916-015-0489-y (PMC4599655; doi:10.1186/s12916-015-0489-y)
Supplement: Additional file 1: Table S6. — Oligonucleotides used for RT-PCR, cloning and Northern blot analyses. (PDF 19 kb) [file 12916_2015_489_MOESM1_ESM.pdf]

SUPPLEMENTAL MATERIAL

Supplemental Tables

Supplemental Table 6. **Oligonucleotides used for RT-PCR, cloning and Northern blot analyses**

	Forward primer	Reverse primer
Mouse and human s-RNY1-5p (RT)		5’-GTCGTATCCAGTGCAGGGTCCGAGGTATTCGCACTGGATACGACATTGAG -3’
mouse s-RNY3-5p (RT)		5’-GTCGTATCCAGTGCAGGGTCCGAGGTATTCGCACTGGATACGACAACACC -3’
human s-RNY3-5p (RT)		5’-GTCGTATCCAGTGCAGGGTCCGAGGTATTCGCACTGGATACGACAGTTGT -3’
human s-RNY4-5p (RT)		5’-GTCGTATCCAGTGCAGGGTCCGAGGTATTCGCACTGGATACGACAGTTCT -3’
U2 snRNA(qPCR)	5’-GAAGTAGGAGTTGGAATAGGA -3’	5’-ACCGTTCCTGGAGGTACTG -3’
s-RNYs universal primer (qPCR)		5’- GTGCAGGGTCCGAGGT -3’
Mouse/human s-RNY1-5p (qPCR)	5’- TGGTCCGAAGGTAGTGAGT -3’	
Mouse s-RNY3-5p (qPCR)	5’- TTGGTCCGAGAGTAG -3’	
Human s-RNY3-5p (qPCR)	5’- TCCGAGTGCAGTGGTGTTT -3’	
Human s-RNY4-5p (qPCR)	5’- GGTCCGATGGTAGTGGGTTAT -3’	
Human RNU48 (qPCR)	5’-AGTGATGATGACCCCAGGTAACTC -3’	5’- CTGCGGTGATGGCATCAG -3’
Mouse and humans s-RNY1-5p (Northern blot)		5’- ACTCACTACCTTCGGACCA -3’
Mouse and humans s-RNY1-3p (Northern blot)		5’- AGTCAAGTGCAGTAGTGAG -3’

mouse 2'-O-Me-RNA antisense to s-RNY1-5p		5'-UUGAGAUAACUCACUACCUUCGGACCAGCC -3'
mouse 2'-O-Me-RNA antisense to s-RNY3-5p		5'-UAAACACCACUACUCUCGGACCAACC -3'
Mouse RNY1 loop (Northern blot)		5'- TTCAATCTGTAACTGACTG -3'
Mouse s-RNY3-5p (Northern blot)		5'- ACCACTACTCTCGGACCAA -3'
Mouse s-RNY3-3p (Northern blot)		5'- CTGGTCAAGTGAAGCAGTG -3'
Human s-RNY4-5p (Northern blot)		5'- CCCACTACCATCGGACCAG -3'
U6 snRNA (Northern blot)		5'-CGTTCCAATTTTAGTATATGTGCTGCCGAAGCGAGCAC-3'
