# Supplementary material for: RNY-derived small RNAs as a signature of coronary artery disease
Source: BMC Med. 2015 Oct 8;13:259. doi: 10.1186/s12916-015-0489-y (PMC4599655; doi:10.1186/s12916-015-0489-y)
Supplement: Additional file 2: Figure S3. — s-RNY expression in apoptotic and lipid-laden macrophages. (A) Northern blot analysis detecting the indicated s-RNYs in human primary macrophages stimulated with 1 μM of staurosporin at the indicated time points. U6 snRNA was used as loading control. (B) Northern blot analysis detecting the indicated s-RNY in bone marrow-derived macrophages (BMDMs) after macrophage-colony stimulating factor withdrawal and reconstitution. (C) Endoplasmic reticulum stress renders macrophages susceptible to apoptosis in the face of other pro-apoptotic stimuli [46]. Quantitative RT-PCR analysis of the indicated s-RNYs in BMDMs incubated for 18 h with 10 mg/mL lipoteichoic acid alone or in combination with 0.25 μM thapsigargin (Tg). The data were normalized by U2 snRNA (n per group = 4). (D) RNA immunoprecipitation analysis of either Ro60 or Argonaute 2 and s-RNY1-5p in BMDMs. RNA was isolated from immunoprecipitation and analyzed by RT-qPCR. BMDMs were left unstimulated or stimulated with 100 μg/mL of oxidized LDL (oxLDL) and 0.25 μM Tg for 28 h. Data are presented as mean and standard deviation (n per group = 3). (E) Northern blot analysis detecting s-RNY1-3p in BMDMs stimulated for 28 h with the indicated concentration of oxLDL in combination with 0.25 μM Tg. U6 snRNA was used as loading control. (F) RT-qPCR analysis of the indicated s-RNYs in BMDMs incubated for 28 h with 0.25 μM Tg in combination with the indicated concentration of acetylated-LDL (acLDL) and control (CTL). The data were normalized by U2 snRNA (n per group = 4). (G) Northern blot analysis using the indicated probes recognizing different parts of RNY1 in BMDMs stimulated with 0.25 mM of palmitic acid for 18 h. U6 snRNA was used as loading control. Student’s t-test: **P <0.01. (PDF 202 kb) [file 12916_2015_489_MOESM2_ESM.pdf]

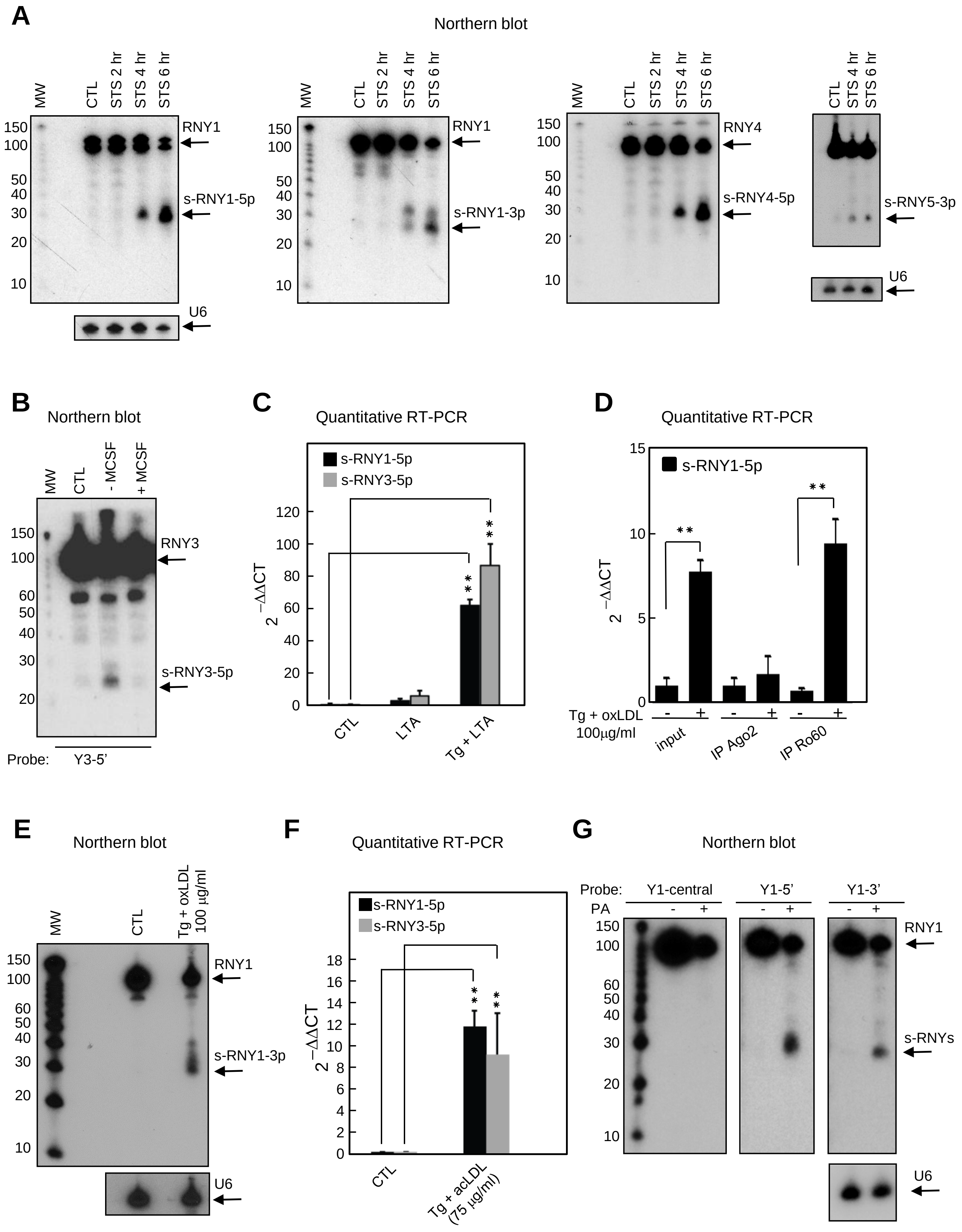

Repetto et al., Supplemental Figure 3
