# Supplementary figures and images for: RNY-derived small RNAs as a signature of coronary artery disease
Source: BMC Med. 2015 Oct 8;13:259. doi: 10.1186/s12916-015-0489-y (PMC4599655; doi:10.1186/s12916-015-0489-y)

**A**

Quantitative RT-PCR

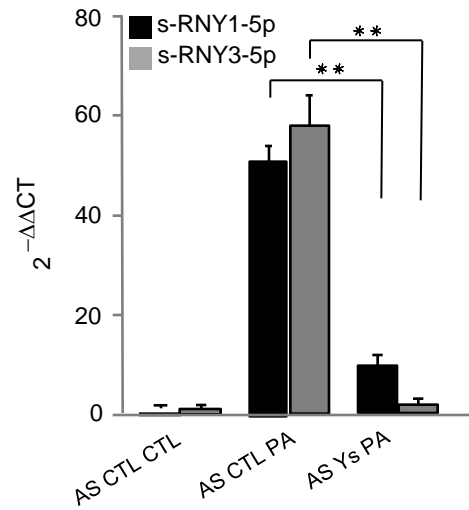**B**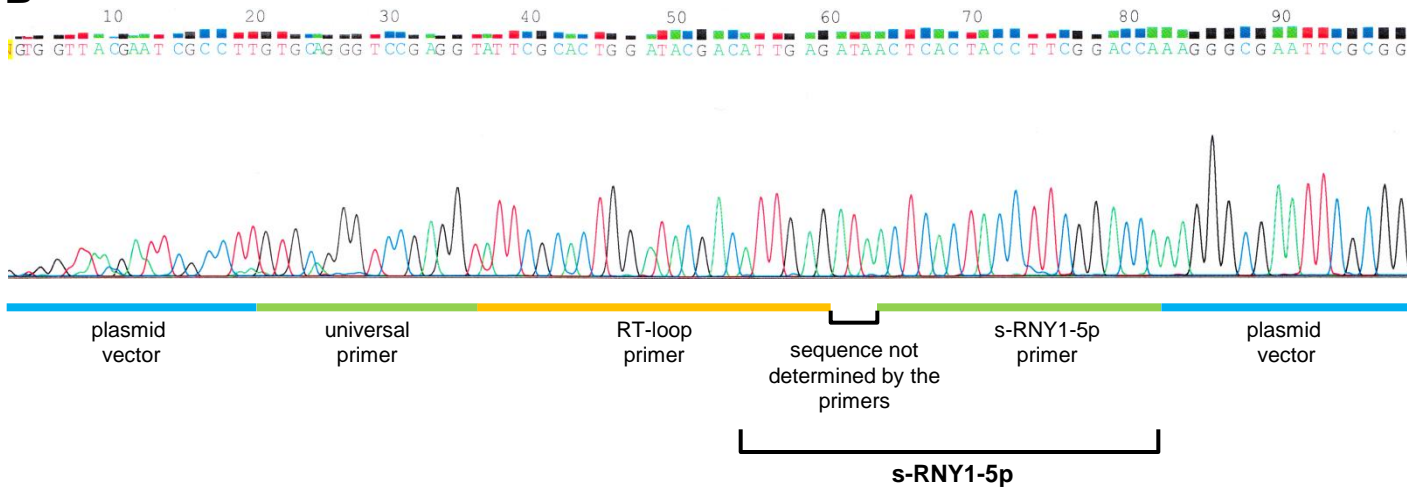

Supplement: Additional file 3: Figure S5. — Control experiments to assess the specificity of the RT-qPCR for s-RNYs. (A) RT-qPCR analysis detecting the indicated s-RNYs in bone marrow-derived macrophages (BMDMs) transfected with 2′-O-Me-RNA antisense oligonucleotides to s-RNYs or control. BMDMs were left unstimulated or stimulated with 0.25 mM of palmitic acid and 0.25 μM thapsigargin for 18 h, and total RNA was isolated and analyzed. Data are presented as mean and standard deviation (n per group = 3), and normalized with the input for the RT-qPCR. (B) Snapshot sequence read from Chromas Lite 2.1 (Technelysium Pty Ltd) showing the RT-PCR product corresponding to s-RNY1-5p from patients with coronary artery disease. Student’s t-test: **P <0.01. (PDF 89 kb) [file 12916_2015_489_MOESM3_ESM.pdf]

**A**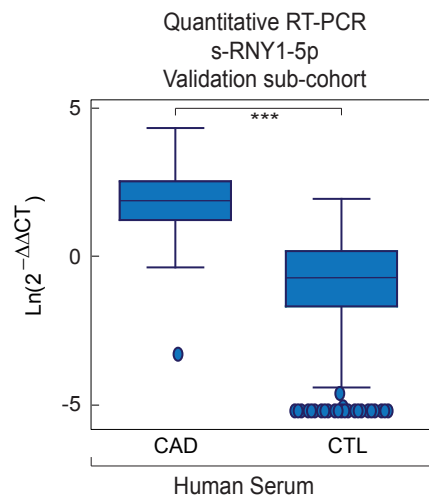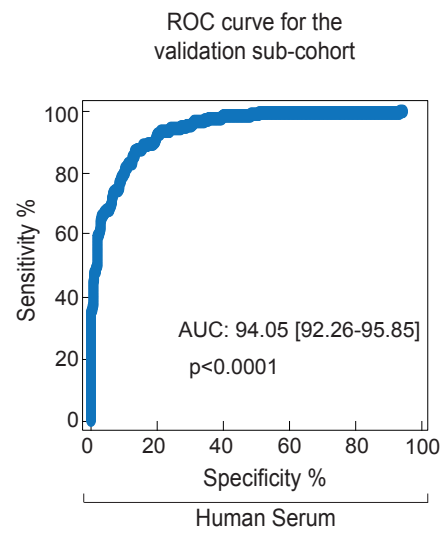**B**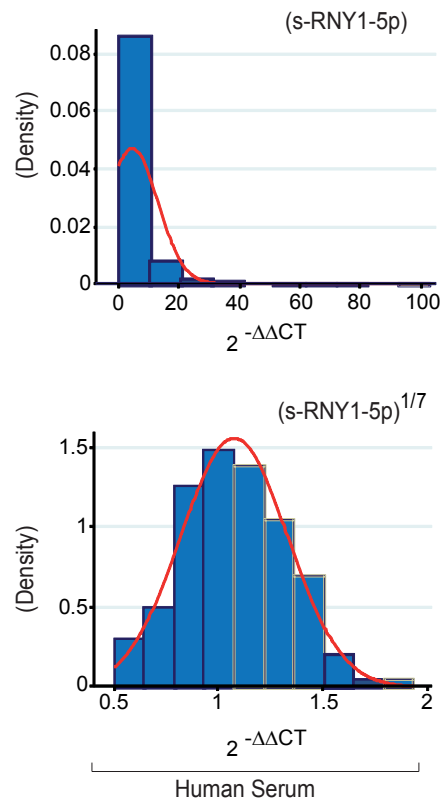**C**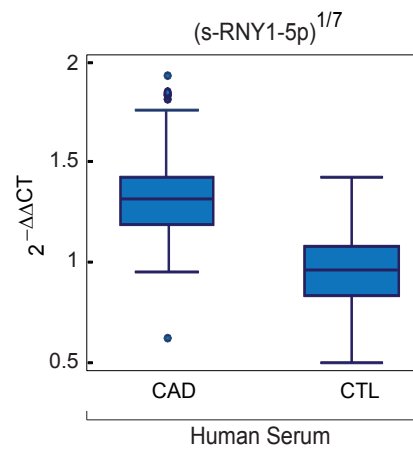

Supplement: Additional file 5: Figure S4. — Validation experiment to assess the specificity of the s-RNY1-5p upregulation in serum of coronary artery disease (CAD) patients compared to healthy control individuals. (A) Box plot showing the expression in natural logarithmic scale of circulating s-RNY1-5p in the validation cohort of 220 CAD patients and 408 controls (left panel). Data derived from RT-qPCR, normalized using cel-miR-39, and presented as mean and standard deviation. Receiver operating characteristic (ROC) curve for predicting CAD with s-RNY1-5p was based on the RT-qPCR data of n = 628 (right panel). The area under the ROC curve for each cohort is indicated. Student’s t-test: ***P <0.0001. (B) Density histogram of s-RNY1-5p levels in the serum of 777 individuals (263 CAD cases and 514 controls) from the GENES study, expressed in crude four values (upper histogram) and values transformed into seventh root (lower histogram). (C) Box plots showing the transformed expression of circulating s-RNY1-5p in the serum of coronary patients (CAD, n = 263) versus healthy control (CTL, n = 514). Data derived from RT-qPCR, normalized using cel-miR-39, and presented as mean and standard deviation. (PDF 387 kb) [file 12916_2015_489_MOESM5_ESM.pdf]

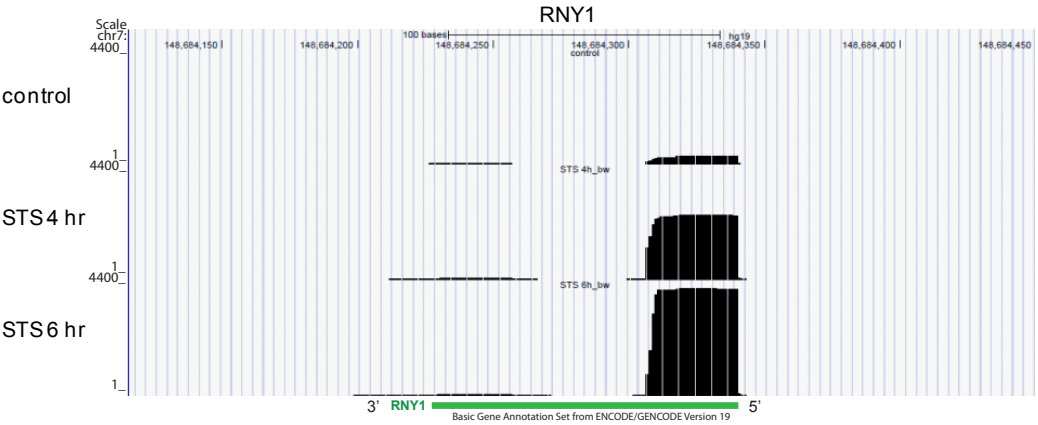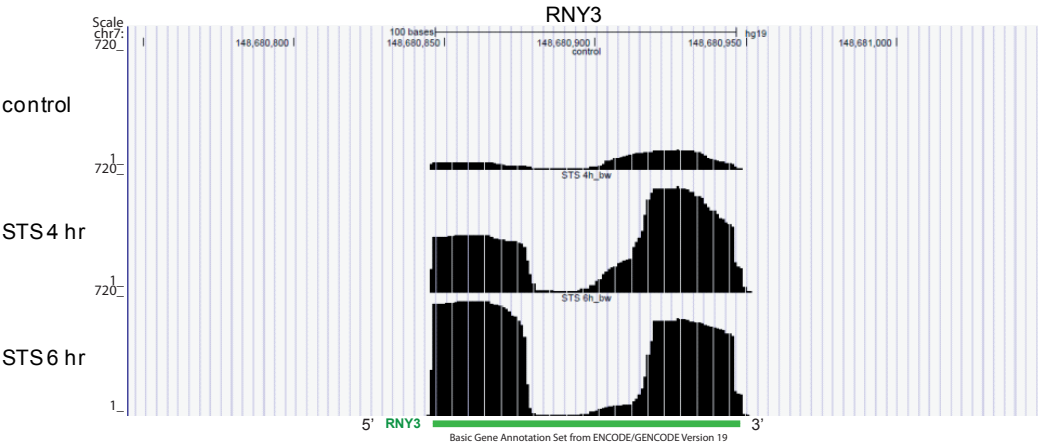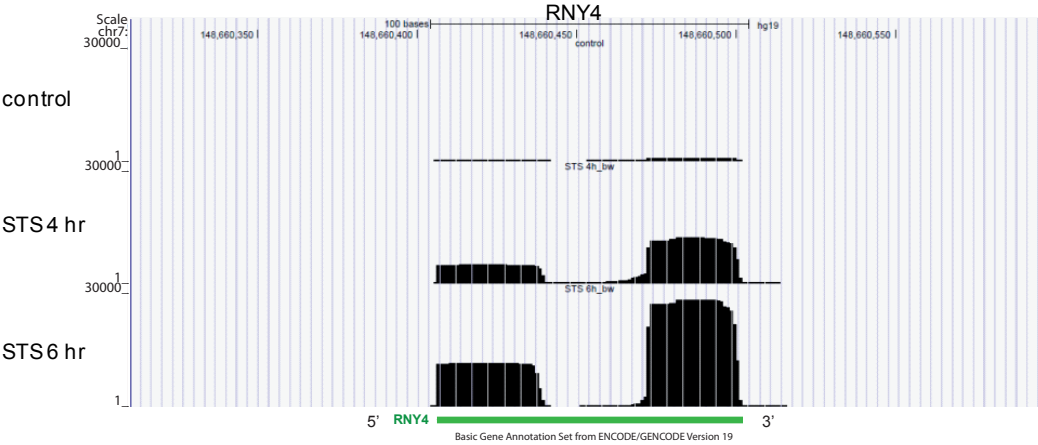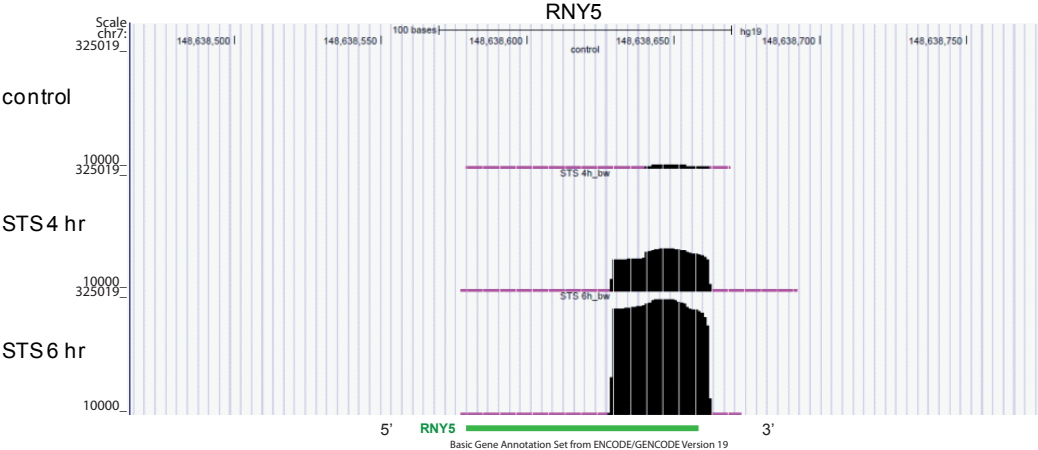

Supplement: Additional file 8: Figure S1. — s-RNY induction in apoptotic human primary macrophages. Bioinformatic analysis of the high-throughput small RNAs sequencing showing the upregulation of small RNA reads derived from RNYs (s-RNYs) in human primary macrophages stimulated with 1 μM of staurosporine at the indicated time points. University of California, Santa Cruz (UCSC) genome browser snapshot encompassing an ~0.2 kb segment of DNA spanning the RNY loci. (PDF 979 kb) [file 12916_2015_489_MOESM8_ESM.pdf]

A

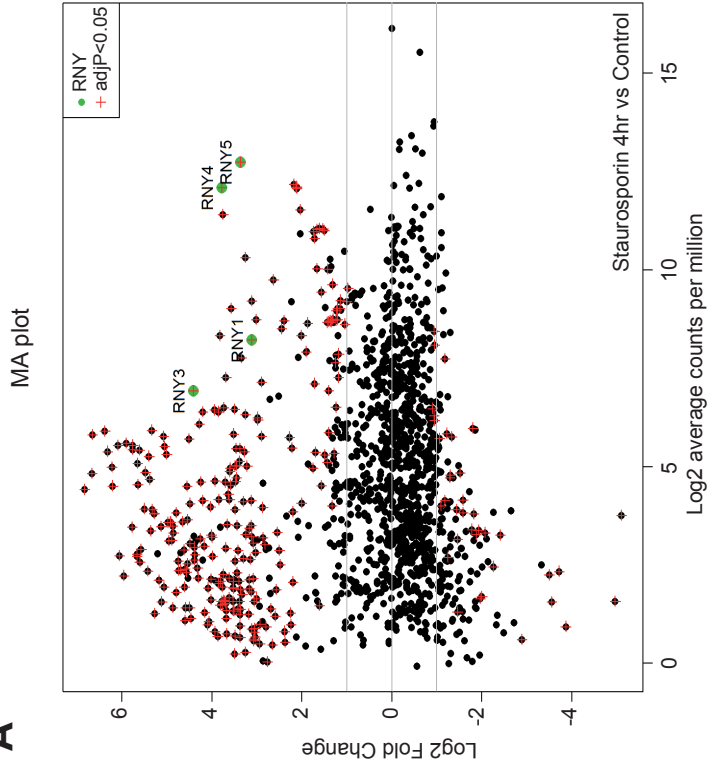

B

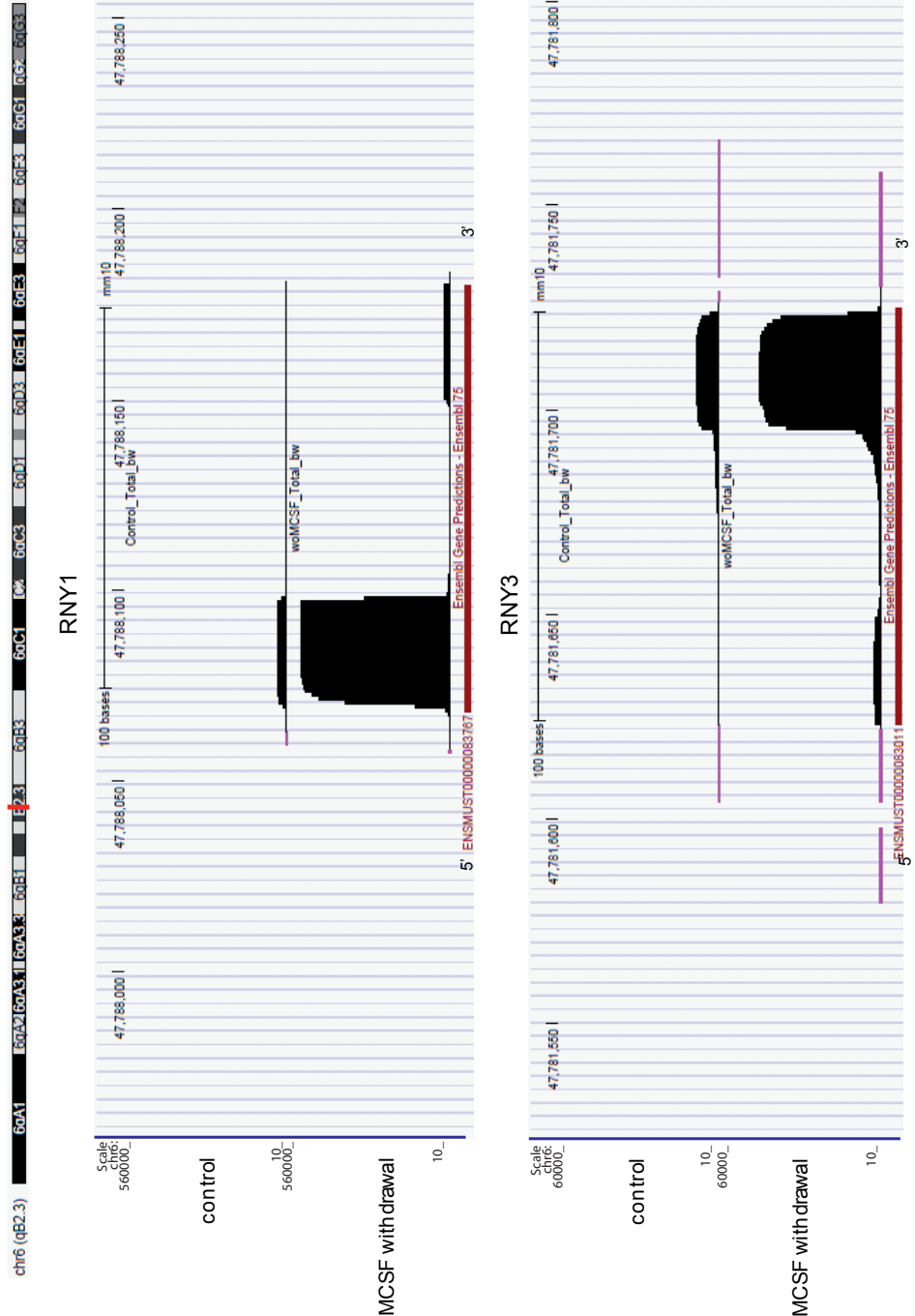

C

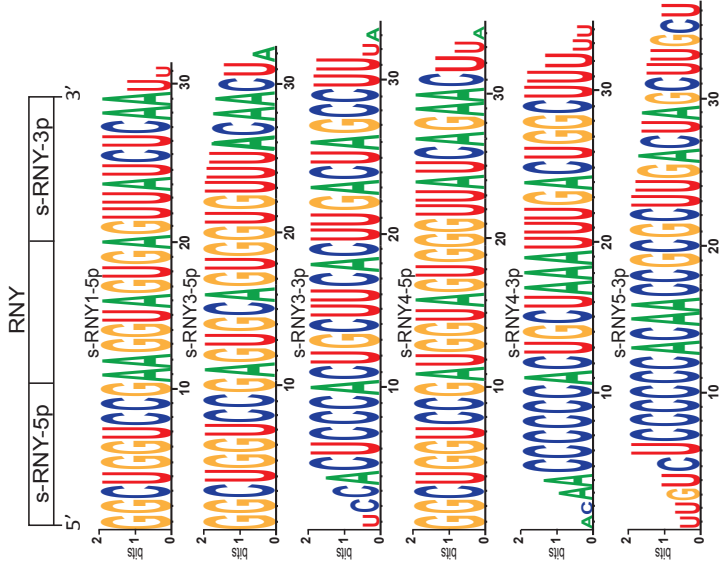

Supplement: Additional file 9: Figure S2. — s-RNY induction in apoptotic mouse primary macrophages. (A) MA plot distribution of differentially expressed small RNAs in human primary macrophages stimulated with 1 μM of staurosporine (STS) for 4 hours compared to control. Green dots indicated RNYs. (B) Bioinformatic analysis of the high-throughput small RNAs sequencing showing the upregulation of small RNA reads derived from RNYs (s-RNYs) in bone marrow-derived macrophages after macrophage-colony stimulating factor withdrawal. University of California, Santa Cruz (UCSC) genome browser snapshot encompassing an ~0.2 kb segment of DNA spanning the RNY loci. (C) Bioinformatic analysis of small RNA-Seq data sets identifying a consensus sequence of s-RNYs in human primary macrophages after STS treatment. The bar graph on the top shows the localization of s-RNYs in RNYs. (PDF 657 kb) [file 12916_2015_489_MOESM9_ESM.pdf]
